# How to differentiate induced pluripotent stem cells into sensory neurons for disease modelling: a functional assessment

**DOI:** 10.1186/s13287-024-03696-2

**Published:** 2024-04-05

**Authors:** Anil Kumar Kalia, Corinna Rösseler, Rafael Granja-Vazquez, Ayesha Ahmad, Joseph J. Pancrazio, Anika Neureiter, Mei Zhang, Daniel Sauter, Irina Vetter, Asa Andersson, Gregory Dussor, Theodore J. Price, Benedict J. Kolber, Vincent Truong, Patrick Walsh, Angelika Lampert

**Affiliations:** 1 Institute of Neurophysiology, Uniklinik RWTH Aachen University, Pauwelsstr. 30, 52074 Aachen, Germany; 2 Research Training Group 2416 MultiSenses-MultiScales, RWTH Aachen University, Aachen, Germany; 3 Center for Advanced Pain Studies, University of Texas at Dallas, Richardson, TX 75080 USA; 4 Department of Bioengineering, University of Texas at Dallas, Richardson, TX 75080 USA; 5 Department of Neuroscience, University of Texas at Dallas, Richardson, TX 75080 USA; 6 Sophion Bioscience Inc., Bedford, MA 01730 USA; 7 Institute for Molecular Bioscience, The University of Queensland, St Lucia, QLD 4072 Australia; 8 School of Pharmacy, The University of Queensland, Woolloongabba, QLD 4102 Australia; 9 Anatomic Incorporated, 2112 Broadway Street NE #135, Minneapolis, MN 55413 USA; 10 Scientific Center for Neuropathic Pain Aachen - SCN-Aachen, Uniklinik RWTH Aachen University, 52074 Aachen, Germany

**Keywords:** Sensory neurons, Disease modelling, Human induced pluripotent stem cells, Sodium channel, Pain

## Abstract

**Background:**

Human induced pluripotent stem cell (iPSC)-derived peripheral sensory neurons present a valuable tool to model human diseases and are a source for applications in drug discovery and regenerative medicine. Clinically, peripheral sensory neuropathies can result in maladies ranging from a complete loss of pain to severe painful neuropathic disorders. Sensory neurons are located in the dorsal root ganglion and are comprised of functionally diverse neuronal types. Low efficiency, reproducibility concerns, variations arising due to genetic factors and time needed to generate functionally mature neuronal populations from iPSCs remain key challenges to study human nociception in vitro. Here, we report a detailed functional characterization of iPSC-derived sensory neurons with an accelerated differentiation protocol (“Anatomic” protocol) compared to the most commonly used small molecule approach (“Chambers” protocol). Anatomic’s commercially available RealDRG™ were further characterized for both functional and expression phenotyping of key nociceptor markers.

**Methods:**

Multiple iPSC clones derived from different reprogramming methods, genetics, age, and somatic cell sources were used to generate sensory neurons. Manual patch clamp was used to functionally characterize both control and patient-derived neurons. High throughput techniques were further used to demonstrate that RealDRGs™ derived from the Anatomic protocol are amenable to high throughput technologies for disease modelling.

**Results:**

The Anatomic protocol rendered a purer culture without the use of mitomycin C to suppress non-neuronal outgrowth, while Chambers differentiations yielded a mix of cell types. Chambers protocol results in predominantly tonic firing when compared to Anatomic protocol. Patient-derived nociceptors displayed higher frequency firing compared to control subject with both, Chambers and Anatomic differentiation approaches, underlining their potential use for clinical phenotyping as a disease-in-a-dish model. RealDRG™ sensory neurons show heterogeneity of nociceptive markers indicating that the cells may be useful as a humanized model system for translational studies.

**Conclusions:**

We validated the efficiency of two differentiation protocols and their potential application for functional assessment and thus understanding the disease mechanisms from patients suffering from pain disorders. We propose that both differentiation methods can be further exploited for understanding mechanisms and development of novel treatments in pain disorders.

**Supplementary Information:**

The online version contains supplementary material available at 10.1186/s13287-024-03696-2.

## Background

Human induced pluripotent stem cell (iPSC)-derived sensory neurons offer a powerful tool to study cellular and molecular mechanisms of human disorders. Like embryonic stem cells, iPSCs are pluripotent, have the potential for self-renewal, and can differentiate into any adult cell type, while they have, in contrast to embryonic cells, the advantage that they can be generated from adult humans with their consent [1]. During embryonic development of vertebrates, neural crest cells (NCCs) originating from the dorsal embryonic neural tube delaminate and migrate into the periphery. These multipotent NCCs further differentiate into several cell types including cells of the peripheral nervous system (PNS), melanocytes, cranial cartilage and bone, neuroendocrine cells, and several other phenotypes [2].

Peripheral sensory neurons with cell bodies located in the dorsal root ganglia (DRG) are a heterogeneous population of neurons that are involved in the detection and transmission of neural signals from the periphery to higher brain areas. Nociceptors respond to noxious thermal, mechanical, and chemical stimuli and can be further classified as A-fibers (myelinated, large fiber and large soma diameter) or C-fibers (unmyelinated, small fiber and small soma diameter). Dysfunction or neurodegeneration of sensory neurons can result in opposite clinical phenotypes ranging from pain insensitivity to severe episodic pain or painful chronic neuropathies [3].

To date, the two most commonly used in vitro model systems for studying cellular and molecular mechanisms of neurological disorders have been rodent primary neurons and heterologous expression systems. Primary rodent neurons originate from non-human species, and, hence, these cells may not recapitulate human physiology and disease pathophysiology sufficiently enough, due to differences in expression of ion channels and receptors mediating pain disorders. Human immortalized lines such as HeLa, HEK293T, CHO, along with neuroblastoma lines such as SH-SY5Y, are easily cultured, provide the possibility for high-throughput analysis, and are scalable. However, they lack, for example, auxiliary Nav β subunits and other proteins which influence excitability, and they do not display a truly neuronal phenotype. So, the use of these immortalized lines for the study of neuron-specific disease mechanisms is limited [4]. Although there is some availability for human tissue [5], to understand human neuronal physiology and neurological disorders, better cellular models than immortalized lines are needed.

Since the successful derivation of sensory neurons from iPSCs by Chambers and colleagues [6], considerable progress has been made in generating neural lineages that can be differentiated into peripheral sensory neurons [7–11]. However, current iPSC differentiation protocols vary widely in efficacy and most likely do not generate the full diversity of nociceptor populations. Differences in the efficacy and reproducibility of differentiation also depend on genetic variations in cell lines, initial density, culture conditions at the start of the experiment, minor differences in small molecules, and coating conditions [12]. It has been reported that small changes in culture conditions and differentiation protocols can significantly change the resulting transcriptome making comparisons between labs and thus reproducibility difficult [13]. It has been shown that iPSC-derived from different somatic cell types result in varied differentiation potential efficiency [14]. These factors remain a key challenge for the generation of subtype-specific neurons from iPSCs. When all these variables are considered, it is often necessary to customize iPSC line specific sensory neuron differentiation protocols to improve yields and efficiencies for different lines grown under different conditions. However, consistent differentiation protocols, which are both highly efficient and rapid, would be beneficial for the generation of functional subtype specific sensory neurons, or a more physiological mixture of subtypes, for human disease modelling.

iPSC-derived from patients suffering from genetic pain syndromes offer the unique possibility to study the effect of genetic variations directly on the affected cells, the patient’s nociceptors [12]. For example, gain-of-function mutations in the SCN9A gene encoding voltage gated sodium ion channel Nav1.7 have been shown to cause Inherited Erythromelalgia (IEM). IEM is a chronic pain condition that results in intense burning pain of extremities often triggered by warming of the affected limbs. Chronic neuropathic pain associated with Small Fiber Neuropathy (SFN) often manifests with intense burning pain in the peripheral limbs and is accompanied by elevated temperature detection thresholds and often by decreased epidermal nerve fiber density [15, 16]. Several mutations have been linked to Nav1.7 and Nav1.8 ion channels causing SFN [17, 18]. This renders voltage-gated sodium ion channels, particularly Nav1.7 and Nav1.8, as attractive therapeutic targets for pain disorders. Unfortunately, most of the selective Nav1.7 and Nav1.8 inhibitors have shown limited clinical efficacy so far [19–21]. iPSC-derived sensory neurons as a disease model system have been used to study IEM [22–24] and SFN disorders [25] providing valuable insight into rare genetic forms of pain. Cao et al. [24] showed the cellular disease phenotype and its reversal through selective Nav1.7 blockade using iPSC-derived sensory neurons from patients with IEM disorder. iPSC-derived sensory neurons helped to identify an FDA-approved drug in vitro as an effective treatment option for a patient suffering from SFN, highlighting the potential of iPSC-derived sensory neurons as a model system to study neurological disorders [25].

Here, we functionally characterize sensory neurons generated from two differentiation protocols: the widely used “Chambers” protocol [6] and the protocol from Anatomic Incorporated (herein referred to as the “Anatomic protocol”) which uses a standard differentiation kit, Chrono™ Senso-DM, inducing a naive early ectodermal intermediate cell type using combined inhibition of bone morphogenic protein and fibroblast growth factor signalling pathways [26]. The Anatomic protocol leads to an accelerated differentiation of iPSCs into immature neurons within Day 7 of differentiation, whereas functional mature neurons emerge from both protocols at Day 28. iPSC-derived from two patients suffering from IEM and SFN chronic pain conditions were used for disease modelling with the Anatomic protocol. Diseased iPSC-derived neurons displayed cellular hyperexcitability and reduced rheobase as compared to control groups, indicating that this protocol may also be used for modelling disease in a dish. RealDRG™ sensory neurons generated using the Anatomic differentiation protocol respond to temperature ramps, indicating the functional presence of heat activated channels such as TRPV1. Automated patch clamp recordings show presence of functional Na^+^ and K^+^ ion channels. Expression studies indicate sensory neurons express SCN10A and TRPV1 nociceptor markers indicating the utility of these neurons for translational pain research. Sensory neurons generated hence provide valuable tool to characterize various ion channels and to facilitate drug discovery and development of novel targets.

## Methods

### iPSC lines from healthy and diseased subjects

In the present study one single clone from four independent lines were selected on the basis of different cell sources, genetics, methodologies used for reprogramming, and that differentiations for comparisons (Anatomic and Chambers Protocol) were attempted once except for Ctrl 1 (healthy subject) line which was differentiated twice in the study. Two control lines were obtained from healthy individuals and two of the lines were obtained from disease-affected patients (Table 1). The timing of neuronal maturation was compared using two different protocols for both of the healthy control lines. In addition, the Anatomic protocol was used to compare the functional properties of sensory neurons obtained from three lines for a comparative study for disease modeling. To demonstrate that sensory neurons derived from the Anatomic protocol are amenable to high throughput technologies for disease modeling and drug discovery applications, the commercially available RealDRG™ sensory neurons were characterized by RNAscope, automated patch clamp, calcium imaging, and multielectrode array (MEA) techniques.

**Table 1:** Clinical phenotype of subjects used in the study

iPSCs	Control/Mutation	Disease condition	Age	Sex	iPS cell source	References
Ctrl1	Healthy control	None reported	69	F	Mesenchymal Stromal cells	[28]
Ctrl2 (Resistant clone)	Healthy control	None reported	6	M	Fibroblasts	[29, 30]
SFN	Nav1.9 (p.N1169S) and Nav1.8 (p.R923H) variant	Small fiber neuropathy	69	F	Fibroblasts	[25]
IEM	Nav1.7 (p.Q875E)	Inherited erythromelalgia	9	F	Blood cells	–
ANAT001 (RealDRG™)	Healthy control	None reported	Neonatal	F	Blood cells (cord blood derived)	–

iPSCs were derived from mesenchymal stromal cells (MSCs) (female 69 years) and fibroblasts (repository name CS00iCTR21n1; Male 6 years) of two healthy subjects designated as Ctrl1 and Ctrl2 respectively (Table 1). Ctrl2 has been referred to as a “resistant clone” in this study due to its lower differentiation potential with Chambers protocol in generating peripheral neuronal lineage (Additional file 1: Fig. S1). iPSC-derived from blood cells of a female 9-year-old suffering from IEM a phenotypically similar but different patient to the first reported by [27] and fibroblasts from a female 69-year-old suffering from SFN disorder (repository name UKERi313-R1, [25] were used for disease modelling designated in this study as IEM and SFN, respectively (Table 1). iPSCs generated from blood cells of the IEM patient were found to be heterozygous for the p.Q875E mutation in the Nav1.7 ion channel (Additional file 1: Fig. S2). iPSCs-derived (ANAT001) from cord blood cells of a healthy subject were used to generate commercially available RealDRG™ (Table 1).

### Reprogramming to iPSCs and maintenance

Peripheral blood mononuclear cells (PBMCs) from the IEM patient were reprogrammed into iPSCs using the CytoTune-iPS 2.0 Sendai Reprogramming Kit (Thermo Fisher Scientific) containing Sendai virus vectors for OCT4, KLF4, SOX2 and c-MYC (Yamanaka factors). IEM iPSCs were maintained on Geltrex (Gibco) in E8 medium prepared in-house (DMEM-F12, E8 supplement, L-glutamine and HEPES) and passaged with 0.5 mM EDTA in PBS every 3–4 days. Reprogramming of mesenchymal stromal cells from healthy subject (Ctrl1) was performed with the plasmids pCXLE-hSK, pCXLE-hUL, pCXLE- hOCT3/4-shp53 transfected by electroporation [28]. iPSCs were cultured on Vitronectin in E8 medium and passaged with 0.5 mM PBS EDTA every 3–4 days. Fibroblasts from another healthy subject (Ctrl2, resistant clone) were reprogrammed into iPSCs by episomal transduction method [29, 30] and Fibroblasts from SFN patient were reprogrammed retrovirally into iPSCs using the Yamanaka factors [25]. Both cell lines were maintained on Vitronectin (Life technologies) in iPSC Brew medium (Miltenyi biotec) and passaged with 0.5 mM EDTA in PBS every 3–4 days. Reprogramming of Ctrl1, Ctrl2, IEM and SFN donor cells was not part of this study. All iPSC clones with a passage number ranging from 11–34 were used in the study. Karyotypes were found to be normal for all the iPSC clones used in the study, further data available on request [25, 28–30]. All iPSC clones used in the study were regularly tested for the mycoplasma contamination.

### iPSC-derived sensory neurons from Anatomic (RealDRG™)

For membrane potential FLIPR assay, multielectrode arrays, automated patch clamp and *in-situ* hybridization studies, Anatomic’s commercially available iPSC-derived sensory neurons (RealDRG™) were used (Table 1). RealDRGs™ were provided as terminally differentiated immature sensory neurons. These neurons were manufactured with scaled-up versions of Anatomic’s Chrono™ Senso-DM kit (Anatomic, cat# 7007). Cord blood cells were reprogrammed using episomal plasmids to generate iPSCs (ANAT001) and were maintained under fully-defined conditions before seeding onto a defined matrix. iPSC clones with a passage number ranging from 10–20 were used in the study. Cultures were fed optimized differentiation formulations daily to produce immature sensory neurons by day 7 post-induction. Day 7 cultures were dissociated and cryopreserved. Lot-specific metrics were recorded including yield, cell number per vial, viability, post-thaw recovery, post-thaw viability, post-thaw morphology, purity, and sterility. Criteria used to determine lots passing quality control included neuronal purity > 95%, verified cell number per vial, post-thaw viability > 70%, and sterility. Lots passing quality control were shipped on dry ice to end users and used according to the manufacturer’s instructions. Additional detail related to differentiation, media compositions, materials used, and bioprocessing steps are proprietary information of Anatomic.

### Informed consent

Informed cosent was obtained for each iPSC clone used in the study. Ctrl1 clone: Femoral bone samples for the isolation of MSCs were collected after informed and written consent, and the study was approved by the Ethics Committee of RWTH Aachen University Medical School (EK 252/12) [28]. Ctrl2 clone: The details of the clone are available in the repository CS00iCTR21n1/GM05400, Coriell [29, 30]. SFN clone: Written informed consent from the patient was obtained having Review Board approvals Nr. 4120 UKER, Germany and Nr. 2012/2297 South East, Norway [25]. IEM clone: Reprograming was performed at Uniklinik RWTH Aachen University with written consent from the patient and her parents (ethics committee of the Medical faculty of the RWTH reference number EK243/18). ANAT001 (RealDRG™) clone: Informed consent was obtained to perform reprogramming of cord blood cells.

### Differentiation of iPSCs to sensory neurons

Differentiation of all iPSCs (healthy and diseased) was carried out using Chrono™ Senso-DM (and Chambers et al. [6] with modifications designated as “Chambers protocol” in this study.

### Anatomic differentiation protocol

The Anatomic differentiation protocol was used for all cell lines investigated in the study. Two variations of the differentiation were compared, one utilizing single cell seeding and one utilizing clump seeding. Ctrl1 iPSCs were differentiated with two different seeding protocols. All other iPSC lines were differentiated with single cell seeding protocol. An optimal seeding density was determined for each cell line with both single cell and clump seeding protocols to maximize yield and efficiency. For clump seeding an ideal iPSC colony size was used ranging between 25 and 50 cells per colony. iPSC cultures with a 60–80% confluence were used for plating the cells as either single cell or clump on Matrix1 (Anatomic, cat# M8001) pre-coated wells. iPSCs were seeded as single cells in a density of 15,000–80,000 cells/cm^2^ with 10 µM Y-27632 (Abcam Biochemicals, Bristol, United Kingdom). For both single cell and clump seeding protocols, a cocktail of small molecules was added from DIV0 (Days in vitro) through DIV7 of differentiation. Chrono™ Senso-DM 1, 2, 3, 4, 5, 6 and 7 (Small molecules, Anatomic Incorporated, cat# 7007) were added on each day of differentiation starting from DIV0. For each day of differentiation, 0.5 mL of Chrono™ Senso DM (1–7) supplement was added to 4.5 mL of Basecamp (Differentiation basal medium, Anatomic) to create 5 mL of complete differentiation medium that was fed immediately to cultures. Immature neurons generated on DIV7 were then dissociated using Accutase (Sigma cat# A6964), incubated at room temperature for ~ 1 h which ensured isolation of single neurons. 30,000–40,000 cells were then plated onto glass coverslips, coated with PDL 0.1 mg/ml (Sigma cat# P0899) and Matrix 3 (Anatomic, cat# M8003). Neurons were then supplemented with Chrono™ Senso-MM maturation medium (Anatomic, cat# 7008) from DIV7 onwards. Two-thirds medium exchanges were performed thrice weekly.

### Chambers differentiation protocol

Ctrl1 cell line was differentiated with the Chambers protocol to compare the potential of differentiation efficiency and generation of sensory neurons to the Anatomic protocol. Ctrl1 iPSCs were differentiated following a previously published protocol with modifications [6, 12]. Briefly, iPSCs were seeded as single cells in a density of 30,000–40,000 cells/cm^2^ with 10 µM Y-27632 (Abcam Biochemicals, Bristol, United Kingdom). When cells reached 80–90% confluency, usually 24–48 h after plating, neural conversion was induced using dual-SMAD inhibition. LDN-193189 1 µM (Sigma-Aldrich) and SB431542 10 µM (Miltenyi Biotec) were added to the culture medium between DIV0-5. To accelerate neural crest specification and peripheral neuron formation from neural crest cells, three small molecules (3 µM CHIR99021, 10 µM DAPT, and 10 µM SU5402, (all Tocris, United Kingdom) were added between DIV2-10. Between DIV0-5, cells were fed with knockout DMEM/F-12 containing 15% KnockOut serum replacement, 1 mM L-glutamine, 100 µM NEAA, 100 µM β-mercaptoethanol, 100 U/ml penicillin and 100 µg/ml streptomycin (all from Thermo Fisher Scientific). Between DIV4-10, cells were fed with DMEM/F-12, containing 10 ml/l N2 (1X), 20 ml/l B27 (1X) without vitamin A supplements and 100 U/ml penicillin, 100 µg/ml streptomycin (all from Thermo Fisher Scientific). N2/B27 medium was added to basal medium at 25% between days 4–5, 50% between days 6–7 and 75% between days 8–10. The culture medium was changed daily.

### Chambers protocol MACS sorting (p75 Neurotrophic receptor)

On DIV10, cells were dissociated using Accutase (Sigma, cat# A6964) and magnetic activated cell sorting (MACS) for CD271 (p75 Neurotrophic receptor) was performed as per the manufacturers protocol (CD271 MicroBead Kit human, MACS Columns and MACS Separators, Neural Crest Stem Cell MicroBeads, human: MicroBeads conjugated to monoclonal antihuman CD271 antibodies all from Miltenyi biotec, MACS buffer 0.5% BSA + 2 mM EDTA). Briefly, cells were dissociated with Accutase for 5 min at 37 °C. The single-cell suspension was then passed through 40 μm cell strainer (Corning, cat# SLS431750) to remove cell clumps. The cell suspension centrifuged at 300 g for 10 min and the cell pellet resuspended into 80 µL of buffer per 10^7^ of total cells. 20 µL of neural crest stem cell microbeads were added (Miltenyi biotec) per 10^7^ total cells and incubated for 15 min in the refrigerator (2–8 °C). Cell suspensions were then applied onto the column and washed 3 times with 500 µL MACS buffer. In the end, magnetically labelled cells were flushed out by firmly pushing the plunger into the column with 1 mL of MACS buffer. 30,000–40,000 cells were seeded onto glass coverslips, coated with 15 µg/ml Poly-L-Ornithine (Sigma, cat# P3655), 10 µg/ml Laminin (Sigma, cat# L2020) and 10 µg/ml fibronectin (Life technologies, cat# 33010018). N2/B27 (Life technologies, cat#17502048 and 17504044) medium supplemented with 20 ng/ml NGF, BDNF, GDNF (all from PeproTech, cat# AF-450-01, 450-02 and 450-10, respectively) and 200 µM ascorbic acid (Sigma, cat# A4544) was used for maturation from day 10 onwards. Medium was changed every 3–4 days. Laminin (500 ng/ml) was added twice weekly in the culture medium.

### Immunocytochemistry

For immunostaining, differentiated neurons were seeded onto glass coverslips, coated with PDL/Matrix3 for Anatomic protocol and Poly-L-Ornithine/Laminin/Fibronectin for Chambers protocol. Cells were fixed with 4% paraformaldehyde and permeabilized and blocked with 5% goat serum (Pan biotechnology, cat# P30-1001) or 1% BSA (Sigma, cat # A9418) and 0.1% Triton X-100 (Sigma, cat# T8787) in PBS (Life technologies, cat# 14190-169). iPSC-derived neurons were then stained with anti-peripherin (Santa Cruz Biotechnology, cat# SC 377093) and anti-β-III-tubulin (TUJ-1) (Cell signaling, cat# 5568S) primary antibodies as a marker for peripherin and neuronal identity respectively. Secondary antibodies were goat anti-rabbit IgG Alexa Fluor 594 (β-III-tubulin) and goat anti-mouse IgG Alexa Fluor 488 (Peripherin). Nuclei were counterstained with DAPI (Thermo Fisher Scientific, cat# SC 377093). Fluorescent images were acquired with a LSM 700 confocal microscope (Carl Zeiss). Fluorescent images were obtained on DIV8-35 and DIV11-35 of freshly generated neurons with Anatomic and Chambers protocol respectively for Ctrl1 and Ctrl2 iPSC-derived neurons. For IEM derived cells peripherin and Tuj1 staining was performed on DIV39. For SFN derived cells peripherin and Tuj1 staining on DIV8 and 14 of freshly manufactured neurons was performed.

### Manual patch clamp recordings

Whole-cell patch-clamp recordings were performed on all four iPSC-derived neurons on DIV14, 21, 28, 35 with both differentiation protocols. Data was pooled for recordings performed over a period of two days for each time point unless specified. Experiments were performed using a HEKA EPC 10 USB amplifier Patch Master and analyzed using FitMaster v2 X 91 software (all HEKA electronics, Lambrecht, Germany), Igor Pro v6.3.7.2 (WaveMetrics, USA) and GraphPad Prism v9.3.1 (GraphPad Software, Inc., La Jolla, USA). Series resistance was compensated by 30–80%. Currents were low pass filtered at 10 kHz and sampled at 100 kHz. Leak current was subtracted using the P/4 method. The liquid junction potential was corrected for both voltage and current clamp recordings. Glass pipettes (Bio-medical Instruments, Zöllnitz, Germany) were pulled with a DMZ puller (Zeitz Instruments, Martinsried, Germany) to a resistance of 1.0 to 3.5 MΩ for voltage clamp recordings and 1.5 to 4 MΩ for current clamp recordings. All experiments were performed at room temperature.

### Current-clamp recordings

Current clamp recordings were performed with extracellular solution containing (in mM): 140 NaCl, 3 KCl, 1 MgCl_2_, 1 CaCl_2_, 10 HEPES, 20 glucose (pH 7.4; 300–310 mOsm) and intracellular solution containing (in mM): 4 NaCl, 135 K-gluconate, 3 MgCl_2_, 5 EGTA, 5 HEPES, 2 Na_2_-ATP, 0.3 Na-GTP (pH 7.25; 290–300 mOsm). Resting membrane potential (RMP) was recorded immediately after establishing the whole-cell configuration for 4 s. The first action potential (AP) evoked by the square pulse protocol (increments of 10 pA) was used to identify the AP properties and maturity of neurons. All recorded neurons having APs with an overshoot above 0 mV were considered mature. The AP threshold was defined as the potential at which the minimum of the first derivative of the AP (the point of inflection during the depolarization) occurs. Afterhyperpolarization (AHP) was calculated as the minimum potential recorded during the repolarization phase of AP. The number of APs generated and time to 1st AP was determined in response to ramp current stimulus of 500 pA/500 ms. Depolarisation current ramps of 1 nA were given over 100–1000 ms to assess firing in response to slow depolarisation. For calculating the number of APs in response to ramp current injections, APs with an overshoot above 0 mV were counted as APs. After measurement in the current-clamp configuration, the amplifier was switched to the whole cell voltage-clamp mode to measure both sodium and potassium currents. Voltage dependence of activation for both Na^+^ and K^+^ currents were recorded with a 500 ms pulse from − 80 mV to 40 mV in 10 mV steps from a holding voltage of − 90 mV.

### Voltage-clamp recordings

To isolate voltage-gated sodium currents, experiments were then performed in the presence of K^+^ and Ca^2+^ ion channel blockers with extracellular solution containing (in mM): 140 NaCl, 1 MgCl_2_, 1 CaCl_2_, 10 HEPES, 1 glucose, 20 TEA-Cl, 1 4-aminopyridine, 0.1 CdCl_2_ (pH 7.4; 300–310 mOsm) and the intracellular solution containing (in mM): 140 CsF, 10 NaCl, 10 HEPES, 1 EGTA, 5 glucose, 5 TEA-Cl (pH 7.3; 290–300 mOsm). Tetrodotoxin resistant (TTXr) currents were recorded in the presence of 500 nM TTX (Tocris Bioscience) diluted in extracellular solution. Voltage dependence of activation of TTXr currents was determined with a 100 ms pulse from − 90 mV to 40 mV in 10 mV increment steps from a holding voltage of − 120 mV.

To assess the presence of Nav1.8 currents, 1 µM of the selective Nav1.8 blocker A-887826 (provided by Grünenthal) was used on DIV31 of maturation from Ctrl1 iPSC-derived sensory neurons [31]. A-887826 was dissolved in DMSO as 10 mM stock solution and the final maximum concentration of DMSO was 0.1%. Solutions were applied through a gravity-driven perfusion system. A single pulse voltage protocol from a holding potential of –120 mV to –30 mV repeated every 10 s was used. Cells were patched in the presence of 500 nM TTX. After obtaining a stable baseline recording with 500 nM TTX, 1 µM A-887826 was applied to check the percent inhibition of currents. In one cell, washout with extracellular solution (ECS) was performed to obtain the total sodium currents at the end of the experiment.

### In situ hybridization

RNAscope in situ hybridization was used to characterize expression of key nociceptor markers in RealDRG™. RealDRG™ iPSC-derived neurons were plated in 8-well chamber slides (Thermo Scientific, cat# 154534) coated with 0.1% PLO (Sigma-Aldrich, cat# P4957)/Matrix 3 (Anatomic) and maintained with Anatomic protocol instructions. In situ hybridization was completed using the RNAscope procedure (multiplex version 1 assay (320851)) utilizing manufacturer’s (Advanced Cell Diagnostics ACD) published protocols [32]. On DIV14 and DIV16, chambers were disassembled from the slide, and the cells were fixed in 10% neutral buffered formalin for 30 min at room temperature. Slides were then washed twice in 1X PBS, and boundaries were drawn around each well using the hydrophobic ImmEdge PAP pen (Vector Labs cat# H-4000). Slides were washed again in 1X PBS before being incubated in protease III reagent (1:30 in 1X PBS) for 10 min at room temperature in a humidity control tray. Slides were washed twice in 1X PBS and then placed in a prewarmed humidity control tray with dampened filter paper to be incubated with probe mixtures for 2 h at 40 °C. DIV14 slides were incubated with Channel 1 *NTRK1* (Neurotrophic Receptor Tyrosine Kinase 1, nociceptor marker gene) (ACD cat# 402631), Channel 2 *TAC1* (Tachykinin precursor 1 codes for neurokinin A and substance P, neuropeptidergic neuronal marker) (ACD, cat# 310711), and Channel 3 *HCN*2 (Hyperpolarization-activated cyclic nucleotide-gated 2 ion channels) (ACD, cat# 517021). DIV16 slides were incubated with Channel 1 *SCN10A* (Sodium ion channel Nav1.8, marker for nociceptors specifically expressed in primary sensory neurons) (ACD, cat# 406291), Channel 2 *TAC1* (ACD, cat# 310711), and Channel 3 *TRPV1* (Transient receptor potential vanilloid subfamily member 1, nociceptor marker gene) (ACD cat# 451381). Slides also had one well each for positive (ACD, cat# 320861) and negative (ACD, cat# 320871) control probes. Following probe incubation, slides were washed twice in 1X RNAscope wash buffer and incubated in AMP-1 reagent for 30 min at 40 °C. Washes and incubation were repeated for AMP-2, AMP-3, and AMP-4A for 15 min, 30 min, and 15 min, respectively. After amplification, slides were washed in 0.1 M phosphate buffer (PB, pH 7.4) and stained with DAPI (Cayman Chemical, cat# 14285). Slides were then washed twice in 0.1 M PB, air dried, and cover-slipped with Prolong Gold Antifade (Fisher Scientific, cat# P36930) mounting medium. Images were acquired on an Olympus FV1200 confocal microscope using a 40X objective and analyzed using Cellsens software (Olympus).

### FLIPR (high-throughput plate reader assays)

For high-throughput fluorescent imaging assays using the FLIPR^Penta^ (Molecular Devices), RealDRG™ were thawed and cultured in Chrono™ Senso-MM Cells were seeded at a density of 1000–10,000 cells/well on black-walled 384-well imaging plates (Corning, cat# CLS3657) pre-coated with Matrix3 and cultured in Anatomic Chrono™ Senso-MM. To record fluorescence responses following stimulation with agonists, cells were loaded with Calcium 4 dye kit (Molecular Devices, cat# R8141) diluted according to the manufacturer’s instructions in physiological salt solution (PSS, composition in mM: NaCl 140, glucose 11.5, KCl 5.9, MgCl_2_ 1.4, NaH_2_PO4 1.2, NaHCO_3_ 5, CaCl_2_ 1.8, HEPES 10) and incubated at 37 °C for 30 min. Responses were measured every 1 s for 300 s and, analysed using ScreenWorks 5.1.1.86 (Molecular Devices).

### Automated Patch clamp recordings

All experiments were performed on Qube384 (Sophion Bioscience A/S). Experiments were executed with single-hole QChips in a format of 48, 120 or 384 simultaneous wells based on the harvest cell numbers. RealDRG™ were thawed and cultured in Chrono™Senso-MM for Automated Patch clamp recordings (APC). Patch clamp recordings were performed on DIV16, 21, 28 and 35 days of maturation. Cells were dissociated using papain (Worthington, cat# LK003150) at 3 units/ml overnight as per the protocol developed by Anatomic. After whole-cell formation controlled with Sophion software, application protocol consisting of both voltage and current clamp protocols were performed at the control condition. Extracellular solution containing (in mM): NaCl 145, CaCl_2_ 2, MgCl_2_ 1, KCl 4, HEPES 10, and Glucose 10, pH 7.4 and intracellular solution containing (in mM): KF 120, KCl 20, HEPES 10, and EGTA 10, pH 7.3 were used for current clamp recordings. To isolate Na^+^ currents, CsF intracellular solution was introduced by using Qube-384 intracellular solution exchange protocol. CsF internal solution contained (mM): CsF 135, NaCl 10, HEPES 10, EGTA 1.0, pH 7.3 with CsOH. 0.5 µM TTX (Alomone Labs) and 1 µM or 10 µM A-803467 (Millipore-Sigma) were used for Na^+^ channel characterization. Cells with a membrane resistance (Rm) > 200 MΩ and a cell capacitance C_slow_ > 2 pF were included in the analysis.

### Multi-well microelectrode arrays

Multi-well microelectrode arrays (MEA) were used to evaluate RealDRG™. The day before starting the culture, a 48-well MEA plate (Axion Biosystems, cat# M768-tMEA-48W) was coated with Poly-L-Ornithine (0.01%, EMD Millipore Sigma, cat#A-004-C) and incubated overnight at room temperature. After 3 washes with sterile deionized water, each well was coated with a Matrix 3 (1:50 dilution with dPBS (-/-) from Anatomic, cat#M8003) and incubated for 3 h at 37 °C. RealDRG™ were thawed and cultured in Chrono™ Senso-MM. Cells were resuspended in 2 mL of Chrono™ Senso-MM complete growth medium and counted for a seeding density of 40 k cells/cm^2^. Immediately prior to seeding, excess Chrono™ Matrix 3 (from plate preparation step) was removed from each well. Cells were added to wells at a total volume of 400 µl in Chrono™ Senso-MM. Chrono™ Senso-MM media was exchanged at 50% from a 400 µl volume every other day and cells were grown at 5% CO_2_ at 37 °C. MEA electrophysiology data were acquired from an Axion Maestro Classic system at 12.5 kHz sampling rate and processed with a single pole Butterworth bandpass filter (300–5000 Hz). Individual spikes were detected from filtered continuous voltage recordings where exceeding ± 5.5σ_RMS_ threshold based on a continuous 1 s data block to estimate σ_RMS_ on a per channel basis. Recordings from the MEA plate were performed for 3 consecutive days each week for 4 weeks after plating. During each session, baseline recordings (at 37 °C) were acquired for a duration of 30 min followed by a temperature ramp up to 42 °C with the embedded heating plate. The time to 42 °C was 2.5 min and typical decay back to 37 °C was 3.5 min. For the sake of classifying cells over time in response to temperature ramps, we grouped recordings into different classes: “Consistent responders”, which includes recordings where the mean firing rate (MFR in Hz) showed elevations from baseline to all 3 temperature ramps for a given week; “responders” were those recordings demonstrating an elevation in at least 1 or 2 of the 3 sessions; “negative responders” were recordings demonstrated spontaneous activity, however there was a decrease in the MFR during temperature ramps; and “inactive electrodes” where the recordings failed to demonstrate spontaneous or temperature ramp-evoked spikes.

### Statistical analysis

Statistical analysis was performed using GraphPad Prism v9.3.1 (GraphPad Software, Inc.). Two groups were compared by a Mann–Whitney or Multiple t tests. Comparisons between three or more groups were performed using a one-Way or two-Way ANOVA followed by Bonferroni’s, Sidak’s or Tukey's multiple comparisons test. Data are presented as mean ± standard error of the mean (SEM) where *P* values < 0.05 were considered significant.

## Results

### Differentiation of iPSCs into sensory neurons

Multiple iPSC lines were used for functional and molecular characterization of sensory neurons to compare the Anatomic differentiation protocol with the small molecule approach [6]. The clinical phenotypes of subjects used in the study are shown in Table 1. iPSCs from all groups were pluripotent-like by morphology as shown in Additional file 1: Fig. S3. These clones were tested for pluripotency markers earlier as described in Namer et al. [25], Hollmann et al. [28], Mattis et al. [29, 30] for SFN, Ctrl1 and Ctrl2, respectively.

**Fig. 1:**
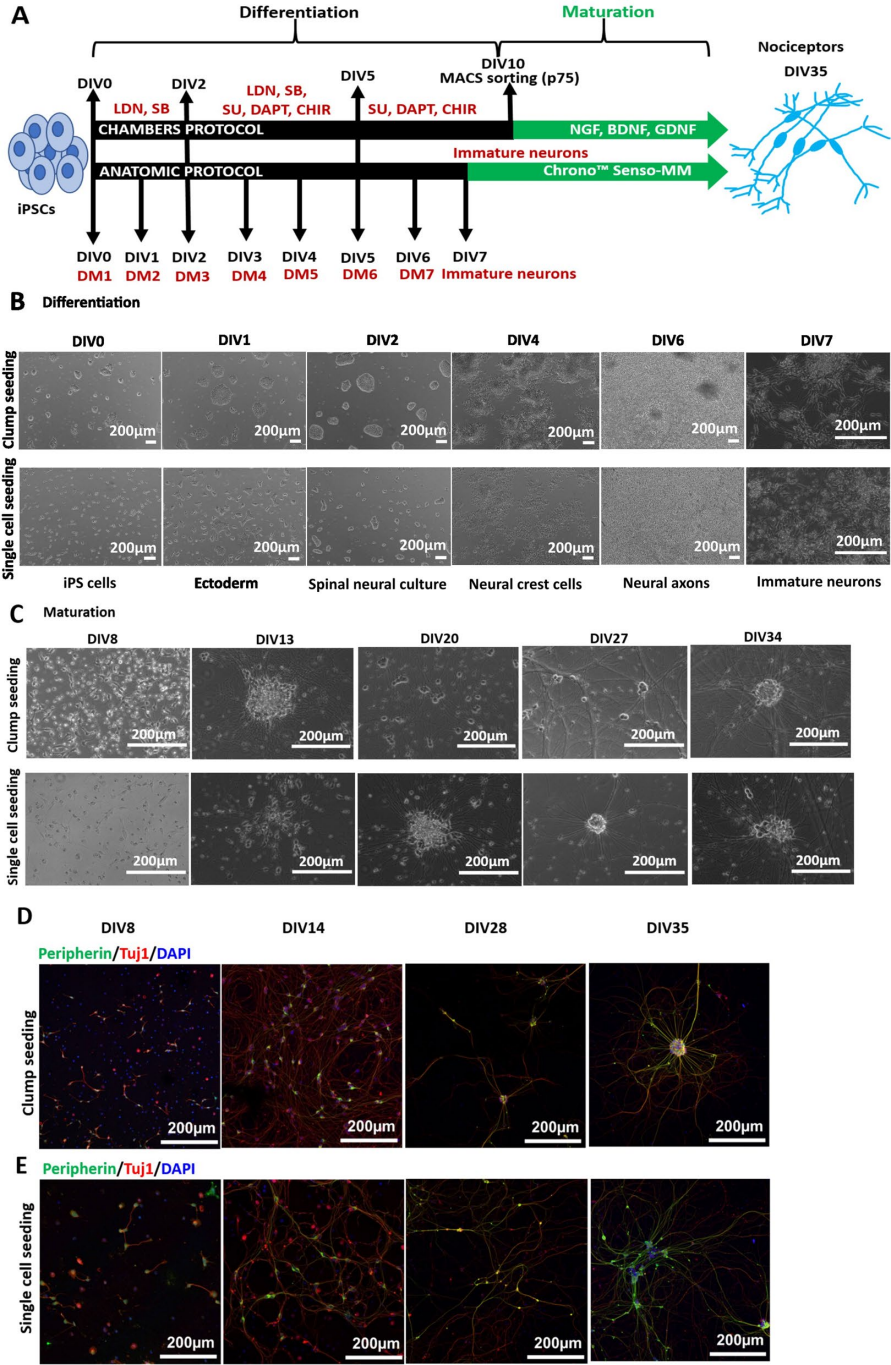
Anatomic protocol: differentiation and maturation (Ctrl1). **A** Schematic diagram outlining the steps for differentiation and maturation with the Anatomic compared to the Chambers protocol. Anatomic protocol requires 7 days of differentiation as compared to 10 days with Chambers protocol to achieve immature neurons. With Chambers protocol neurons were MACS sorted on DIV10 of differentiation. Neurons were then matured until DIV 35–40. DIV-Days in vitro, LDN-193189, SB431542 and SU5402, CHIR99021 and DAPT. DM-Differentiation medium, Chrono™ Senso-MM-Maturation medium. **B** Differentiation of Ctrl1 iPSCs with Anatomic protocol. Phase contrast images display single cell and clump seeding on DIV0 of differentiation. Differentiation involves formation of ectoderm within 24 h, spinal neural culture (DIV2), neural crest formation (DIV4) and generation of immature neurons by DIV7. Scale Bar—200 µm. **C** Maturation of Ctrl1 neurons with growth factors. Both seeding protocols resulted in formation of dense homogenous neuronal networks during the maturation period. No morphological differences could be observed during maturation from both protocols. Scale Bar—200 µm. **D** Immunostaining of neurons for Peripherin and Tuj1 on DIV8, 14, 28 and 35 with clump seeding. **E** Single cell seeded neurons staining for Peripherin and Tuj1 on DIV8, 14, 28 and 35. Scale Bar—200 µm. Peripherin-green, Tuj1-red and DAPI-blue fluorescence. DIV-Days in vitro

All iPSC clones were differentiated into peripheral neurons using the Anatomic protocol for 7 days followed by feeding the culture with Anatomic maturation medium until 4–5 weeks. Ctrl1 iPSCs were additionally differentiated with the Chambers protocol for 10 days and MACS sorted for p75 NTR marker followed by maturation for 4 weeks. The schematic outlining of both the protocols is shown in Fig. 1A.

### Sensory neurons derived from single cell or clump seeded iPSCs show similar functional properties

Human embryonic stem cells (ESCs) and iPSCs survive poorly after individualization (i.e., dissociation and reseeding as single cells) because these cells are more sensitive to treatments and are prone to cell death [33]. Stem cells are usually passaged as aggregates during enzymatic dissociation which allows for a long-term expansion without affecting the cell survival and karyotype [34]. Most of the differentiation protocols reported in the literature involve single-cell seeding of iPSCs for generation of sensory neurons. Single cell maintenance requires using a ROCK inhibitor to enhance cell survival as longer exposure has shown changes in cellular metabolism [35]. We assessed the differentiation potential of the Anatomic protocol with single cell versus clump seeding methods. Seeding at too low density can result in substantial loss of viability. Very large iPSC colonies (200 cells or greater) will affect downstream differentiation processes. We optimized the seeding densities for maximum efficiency.

iPSCs from Ctrl1 were used to investigate if the two seeding methods result in differences in the efficiency to produce neurons, expression for peripheral neuronal marker proteins, and in their resulting AP properties. Phase contrast imaging of the differentiation DIV0 to DIV7 did not suggest any morphological differences between single cell and clump seeding methods (Fig. 1B). Cells were further grown in maturation medium until DIV39. Figure 1C shows phase contrast images of neuronal maturation from DIV8 to DIV34. iPSCs from Ctrl2 were also differentiated with both seeding protocols with comparable morphological appearance to Ctrl1 (Additional file 1: Fig. S4).

Immunofluorescence staining across different time points DIV8, 14, 28 and 35 was performed. We checked for Peripherin/Tuj1 marker expression to confirm peripheral lineage of generated neurons. Immunostaining confirmed expression of peripherin and Tuj1 proteins at DIV8, 14, 28 and 35 of maturation with both the seeding methods (Fig. 1D, E).

**Fig. 2:**
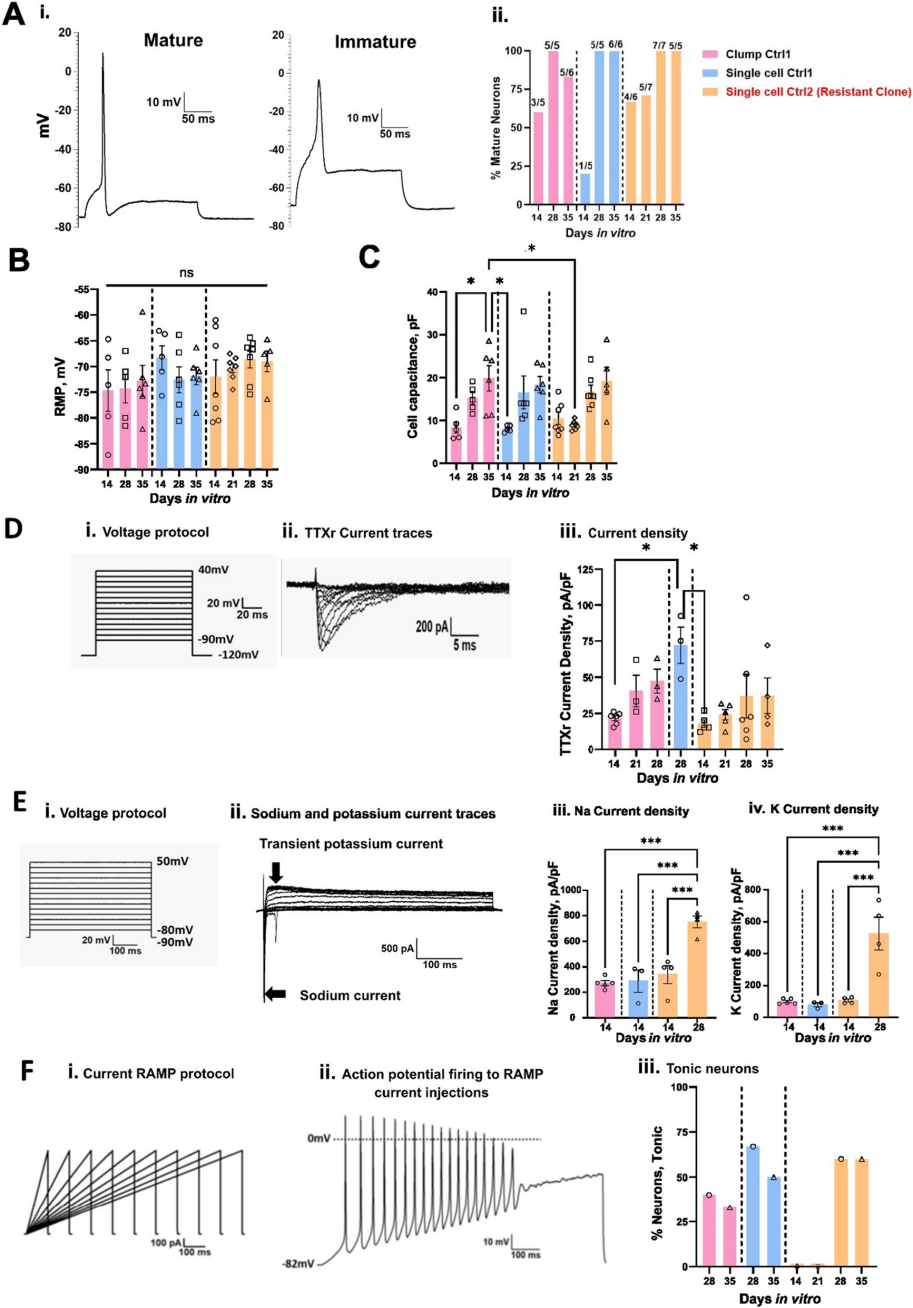
Electrophysiological characterization of Anatomic-derived neurons (Ctrl1 and Ctrl2). **A** Comparison of percentage mature neurons (first AP generated with a square pulse current injection with steps of 10pA) between clump and single cell seeding. i. Representative mature and immature AP traces ii. Percentage of mature neurons: Clump Ctrl1- DIV14: 60% (n = 3 out of 5), DIV28: 100% (n = 5 out of 5), DIV35: 83% (n = 5 out of 6) and Single cell Ctrl1- DIV14: 20% (n = 1 out of 5), DIV28: 100% (n = 5 out of 5), DIV35: 100% (n = 6 out of 6). Ctrl2—Percentage of mature neurons: DIV14: (67% (n = 4 out of 6), DIV21: 71% (n = 5 out of 7), DIV28: 100% (n = 7 out of 7) and DIV35: 100% (n = 5 out of 5). Number of mature cells out of total cells patched are denoted for each recording day above the bar. **B** RMP of Ctrl1 and Ctrl2 neurons. **C** Cell capacitance (pF) for Ctrl1 and Ctrl2 neurons. **D** TTXr currents recorded in the presence of 500 nM TTX. i. Voltage protocol, ii. Representative current traces, iii. Current density measured on DIV14, 28 and 35. **E** Voltage clamp recordings of inward sodium and outward potassium currents. i. Voltage protocol ii. Na^+^ and K^+^ current traces. iii. Sodium current density measured on DIV14 and 28 of maturation. iv. Potassium current density measured on DIV14 and 28 of maturation. **F** Tonic firing in response to ramp current stimuli i. Ramp current stimuli, ii. AP firing in response to ramp current stimuli 1nA/1 s. iii Percentage tonic neurons. Data shown as mean ± SEM. One-way Anova Bonferroni's multiple comparisons test

Neurons were classified as mature or immature based on their ability to generate over-shooting APs [36]. We examined the electrophysiological properties of these neurons at DIV14, 28 and 35 and divided the APs either as mature or immature based on spike overshoot, where mature was defined as showing an overshoot above 0 mV. We found that immature neurons revealed a wider half-width duration as compared to mature neurons as shown in (Fig. 2A i). We found 100% of neurons patched display mature APs starting at DIV28 of maturation for both clump and single cell seeding methods (Fig. 2A ii). The RMP did not show any significant differences over the maturation time (Clump Ctrl1 DIV14 − 74.7 ± 4 mV n = 5, DIV28 − 74.3 ± 2.8 mV n = 5, DIV35 − 72.8 ± 3 mV n = 6, Single cell Ctrl1 DIV14 − 68.3 ± 2.3 mV n = 5, DIV28 − 72.6 ± 2.5 mV n = 6, DIV35 − 71.9 ± 1.7 mV n = 6 *p* > 0.05) (Fig. 2B and Table 2). Cell capacitance increased with longer maturation time for both single cell and clump seeding protocols, but no significant difference was observed between the groups at corresponding days of maturation (Fig. 2C and Table 2).

**Table 2:** AP parameters Ctrl1 and Ctrl2

Parameters	Clump Ctrl1			Single cell Ctrl1			Single cell Ctrl2 (Resistant clone)			
	DIV14	DIV28	DIV35	DIV14	DIV28	DIV35	DIV14	DIV21	DIV28	DIV35
RMP, mV	− 74.7 ± 4.0 (5)	− 74.3 ± 2.8 (5)	− 72.8 ± 3.0 (6)	− 68.3 ± 2.3 (5)	− 72.6 ± 2.5 (6)	− 71.9 ± 1.7 (6)	− 72.0 ± 3.3 (7)	− 70.3 ± 0.9 (7)	− 68.6 ± 1.6 (7)	− 69.0 ± 2.0 (5)
Cell capacitance, pF	8.4 ± 1.3 (5)	15.2 ± 1.4 (5)	19.8 ± 3.0^a^ (6)	8.0 ± 0.3^b^ (5)	16.5 ± 3.8 (6)	18.3 ± 1.9 (6)	10.5 ± 1.4 (7)	8.9 ± 0.4^c^ (7)	16.7 ± 1.5 (7)	19.2 ± 3.2 (7)

Data as Mean ± SEM

^a^*p* < 0.05 versus DIV 14 Clump Ctrl1

^b^*p* < 0.05 versus DIV 35 Clump Ctrl1, ^c^*p* < 0.05 versus DIV 35 Clump Ctrl1

The values in brackets indicate the sample number, *DIV* Days in vitro

The SCN10A gene encoding the TTXr voltage-gated ion channel Nav1.8 has been functionally characterized in both rodent and human DRG neurons [37]. To test for its functional expression, we performed whole-cell voltage-clamp recordings with 500 nM TTX. Voltage protocol and representative TTXr current traces are shown in Fig. 2D i and ii respectively. We found an increase in TTXr current density with maturation time for Ctrl1 (clump seeding) neurons (Fig. 2D iii and Table 3).

**Table 3:** TTXr current density Ctrl1 and Ctrl2

Parameter	Clump Ctrl1			Single cell Ctrl1	Single cell Ctrl2 (Resistant clone)			
	DIV14	DIV21	DIV28	DIV28	DIV14	DIV21	DIV28	DIV35
TTXr current density, pA/pF	21.7^a^ ± 1.7 (6)	40.6 ± 10.9 (3)	47.4 ± 8.3 (3)	72.2 ± 12.7 (3)	18.0^b^ ± 2.6 (5)	24.1 ± 3.6 (5)	36.9 ± 15.1 (6)	37.3 ± 12.3 (4)

Data as Mean ± SEM

^a^*p* < 0.05 versus DIV28 Single cell Ctrl1

^b^*p* < 0.05 versus DIV28 Single cell Ctrl1

The values in brackets indicate the sample number, DIV—Days in vitro

A-887826 blocked TTXr Na^+^ currents from rat DRG neurons in a state-dependent fashion with an IC_50_ of 7.9 ± 0.2 nM for the inactivated state (− 40 mV) to an IC_50_ of 63.6 ± 0.2 nM when channels were held in a resting state at − 100 mV [31]. To identify the functional Nav1.8 ion channel expression in the generated neurons, we perfused 1 µM A-887826 in the presence of 500 nM TTX on iPSC-derived sensory neurons at DIV31 of maturation. A single pulse voltage clamp protocol was used from holding potential of − 120 mV to − 30 mV, applied every 10 s (Additional file 1: Fig. S5 i). A-887826 inhibited more than 95% of TTXr currents (n = 2) suggesting the presence of TTXr Nav1.8 currents in the iPSC-derived nociceptors (Additional file 1: Fig. S5 ii).

We further evaluated the functional expression of sodium and potassium ion channels using physiological solutions and performed voltage clamp recordings on DIV14 of maturation. When held at -90 mV, both inward Na^+^ and outward K^+^ currents were recorded, indicating functional expression of both Na^+^ and K^+^ ion channels in these sensory neurons (Additional file 1: Fig. 2E i and ii). No significant difference was observed in peak Na^+^ (Clump Ctrl1 at DIV14: 271.2 ± 19.2, n = 5; Single cell Ctrl1: 288 ± 87.5, n = 3, *p* ≥ 0.9) or K^+^ (Clump Ctrl1 DIV14 99.2 ± 7.5 n = 5, Single cell Ctrl1 79.5 ± 12.8 n = 3 *p* ≥ 0.9) current densities (pA/pF) between single cell and clump seeding methods (Fig. 2E iii–iv and Table 4).

**Table 4:** Sodium and potassium currents Ctrl1 and Ctrl2

Parameters	Clump Ctrl1	Single cell Ctrl1	Single cell Ctrl2 (Resistant clone)	
	DIV14	DIV14	DIV14	DIV28
Na^+^ current density, pA/pF	271.2 ± 19.2* (5)	288.0 ± 87.5* (3)	339.4 ± 69.9* (4)	752.1 ± 46.5 (4)
K^+^ current density, pA/pF	99.2 ± 7.5^a^ (5)	79.5 ± 12.8^a^ (3)	106.3 ± 9.6^a^ (4)	525.5 ± 102.6 (4)

Data as Mean ± SEM

*p* < 0.05 versus DIV 28 Single cell Ctrl2 (Resistant clone)

^a^*p* < 0.05 versus DIV 28 Single cell Ctrl2 (Resistant clone)

The values in brackets indicate the sample number, DIV—Days in vitro

We applied depolarizing 1 nA ramp currents over durations ranging from 100 to 1000 ms to assess firing in response to slower depolarization changes (Fig. 2F i) and classified these neurons as tonic or phasic firing. Neurons were considered tonically firing if they generated ≥ 2 APs and phasic for single AP only. Representative AP firing in response to ramp stimuli (1 nA/ s) are shown in Fig. 2F ii. We observed tonic firing neurons for both Ctrl1 and Ctrl2 neurons on DIV28 and DIV35 of maturation, Clump seeding DIV28: n = 5 (2/5 Tonic), DIV35: n = 6 (2/6 Tonic), Single cell DIV28: n = 3 (2/3 Tonic), 1 cell did not generate any AP, DIV35: n = 6 (3/6 Tonic). For Ctrl2 clone, Percentage tonic neurons DIV14: n = 5 (0/5 Tonic), DIV21 n = 6 (0/6 Tonic), DIV28 n = 5 (3/5 Tonic). DIV35 (n = 5 3/5 Tonic) (Fig. 2F iii).

These data suggest that both single cell and clump seeding protocols result in generation of peripheral sensory neurons with almost no difference in the morphological, electrophysiological and marker expression properties of generated neurons. We thus performed further differentiations of all clones used in the study with the single cell seeding protocol.

### Anatomic protocol allows the differentiation of a “resistant” iPSC clone derived from a healthy subject

Differentiation of iPSCs into sensory neurons, especially when using the Chambers differentiation protocol, suffers from high clone-to-clone variability [12]. We have identified a specific iPSC clone derived from a healthy subject (Ctrl2 termed as “resistant clone”), which does not differentiate well with the Chambers protocol. With the Chambers protocol, differentiation results in neuronal cultures with a very low percentage of peripherin-positive neurons (Additional file 1: Fig. S1). To understand if the Anatomic protocol results in improved differentiation and more effective generation of sensory neurons with a resistant clone like Ctrl2, we performed a detailed functional and immunocytochemistry analysis on Ctrl2 iPSC-derived neurons.

**Fig. 3:**
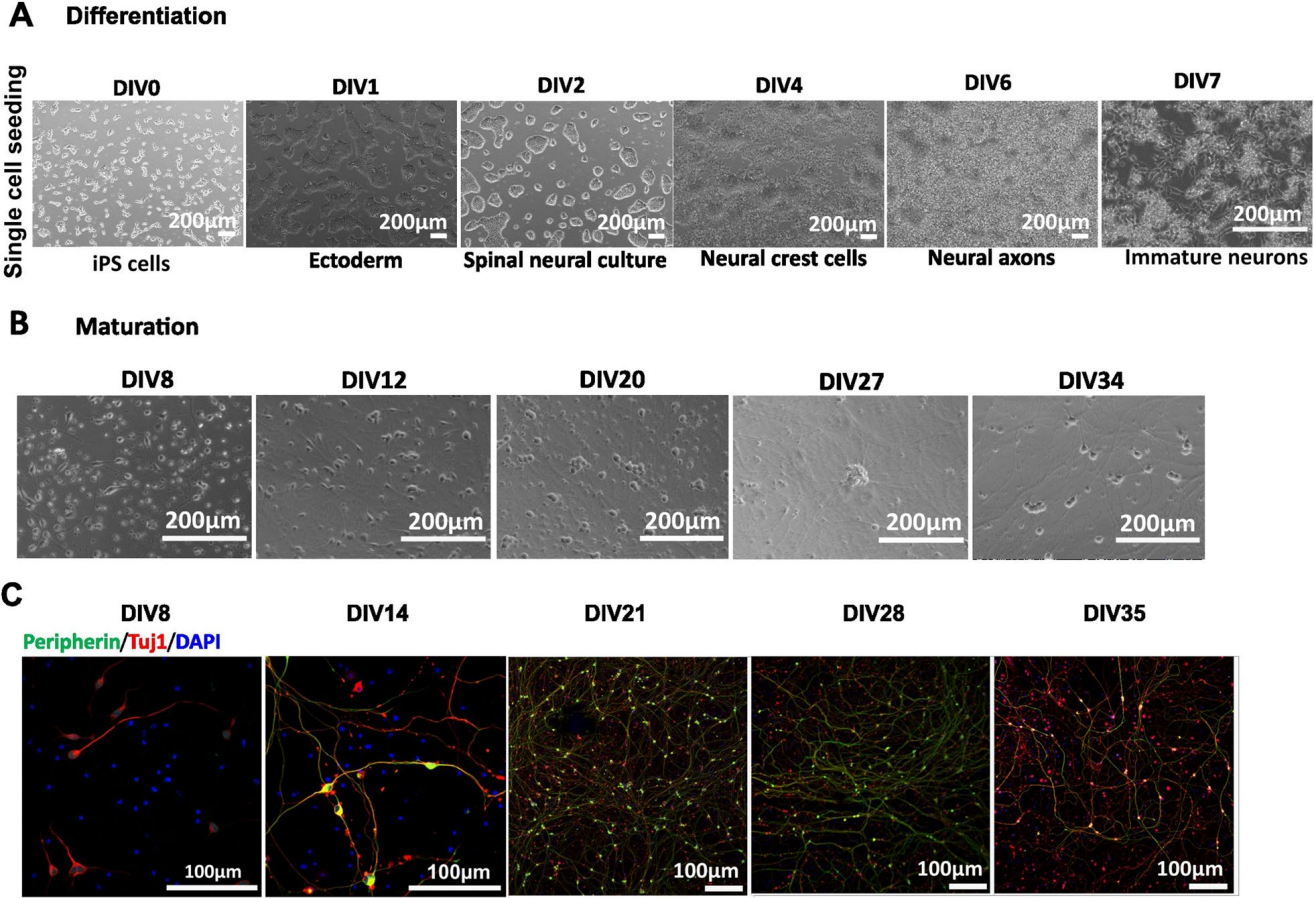
Anatomic protocol: Differentiation and Maturation (Ctrl2). **A** Differentiation: Phase contrast images display single cell seeding of iPSCs on DIV0 of differentiation and generation of immature neurons by DIV7. Scale bar—200 µm. **B** Maturation: Maturation of neurons DIV7-35. Differentiation resulted in a homogenous neuronal network during the maturation period. Scale Bar 200 µm. **C** Immunostaining of neurons for Peripherin and Tuj1 on DIV8, 14, 21, 28 and 35. Scale Bar—100 µm. DIV-Days in vitro. Peripherin-green, Tuj1-red and DAPI-blue fluorescence

Phase contrast imaging of the differentiation DIV0 to DIV7 shows generation of immature sensory neurons by DIV7 (Fig. 3A). Cells were further cultured in maturation medium until DIV35. Figure 3B shows phase contrast images of neuronal maturation from DIV8 to DIV34. Immunostaining confirmed the marker expression of peripherin and Tuj1 proteins from DIV8 onwards in these cells (Fig. 3C).

To further confirm the functional properties of these neurons, we performed patch clamp recordings on different days of maturation. Mature APs were detected starting at DIV14, and their percentage increased with maturation time (Fig. 2A ii). RMP was not significantly different from DIV14 to 35 (Fig. 2B and Table 2). A significant increase in cell size was observed with maturation similar to Ctrl1 iPSC-derived neurons (Fig. 2C and Table 2). Voltage clamp recordings suggest the functional expression of TTXr currents, with an increase in current density recorded from DIV14 to DIV35 (Fig. 2D iii and Table 3). We found a significant increase in the Na^+^ and K^+^ current density from DIV14 to DIV28 (Fig. 2F iii–iv and Table 4). Using ramp stimulation to identify tonic and phasic firing neurons, we observed tonic firing neurons starting DIV28 for Ctrl2 neurons (Fig. 2G iii).

These data suggest that the Ctrl2 iPS cell line, which was resistant to differentiate with the Chambers protocol, displayed robust differentiation towards a peripheral neuronal lineage and generates a dense neuronal network of sensory neurons with the Anatomic protocol.

### Chambers protocol results in predominantly tonic firing neurons compared to Anatomic protocol

To understand the functional and immunocytochemical differences between the Anatomic and Chambers protocols, we differentiated blood-derived iPSCs from a healthy subject (Ctrl1) into sensory neurons using both protocols.

**Fig. 4:**
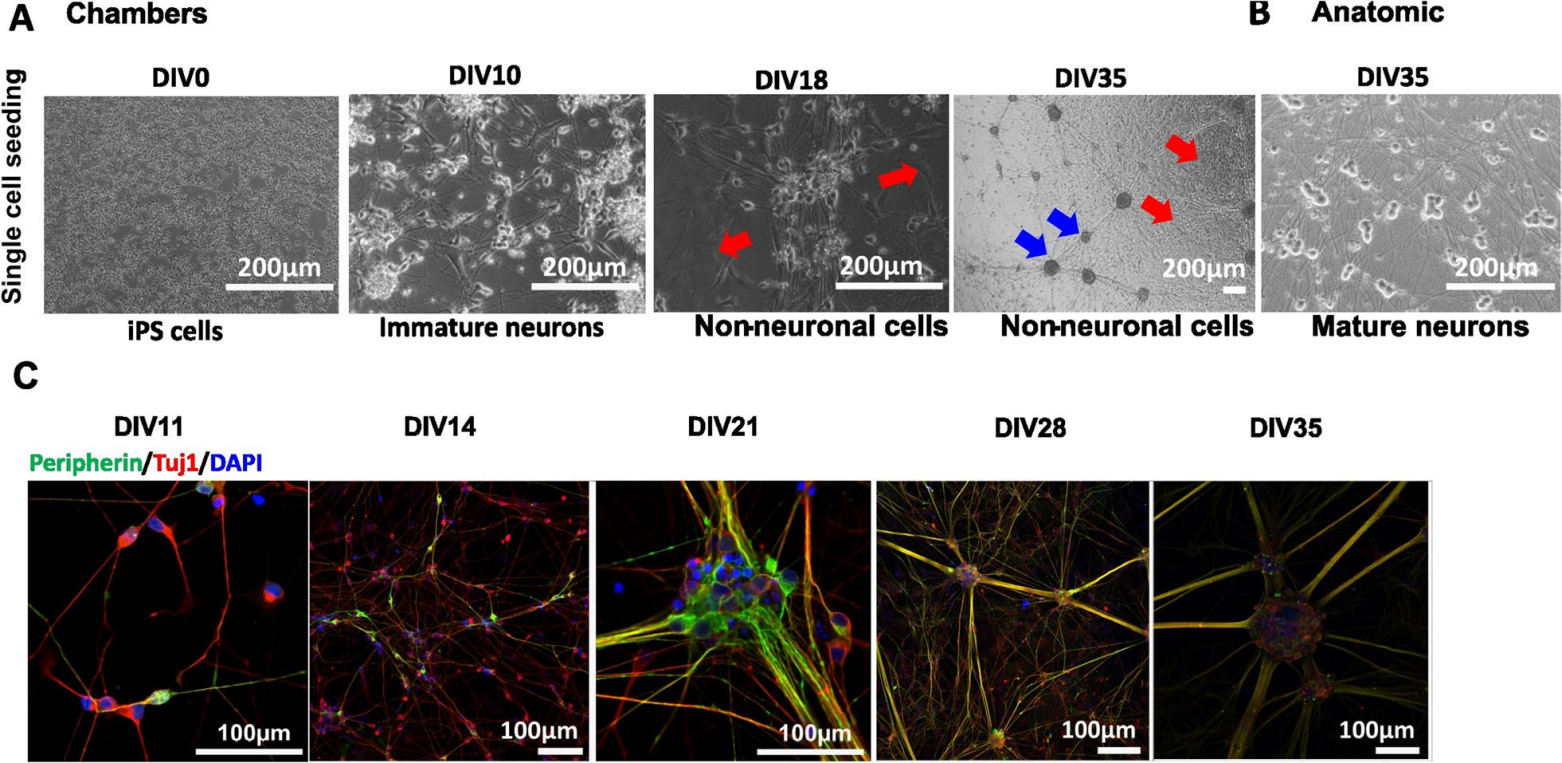
Chambers protocol: Differentiation and Maturation (Ctrl1). **A** Phase contrast images during the differentiation and maturation of iPSC-derived from Ctrl1 subject. DIV0 iPSCs, DIV10 generation of immature neurons, DIV35 shows ganglion-like morphology (marked with blue arrows). DIV18 and 35 also shows the culture outgrown with other non-neuronal cell types (marked with red arrows). Scale bar—200 µm. **B** Neurons at DIV35 from Anatomic protocol results in homogenous neuronal network. Scale bar—200 µm. **C** Immunostaining confirms peripheral neuronal identity of neurons from DIV11, 14, 21, 28 and 35 of maturation. DIV-Days in vitro. Scale bar − 100 µm. Peripherin-green, Tuj1-red and DAPI-blue fluorescence

Phase contrast imaging shows seeding of Ctrl1 single cells on DIV0 and formation of immature neurons by DIV10 of differentiation with Chambers protocol (Fig. 4A). To enrich the neuronal population towards homogeneous peripheral neuronal cultures, we performed MACS sorting for CD271-expressing cells on DIV10 of differentiation. Neural progenitors obtained were then matured until DIV35. We found significant growth of non-neuronal cells as shown in Fig. 4A at DIV18 and DIV35 marked with red arrows, respectively. Young et al. [8] reported the use of antimitotic agents to reduce the number of proliferating non-neuronal cells at the beginning of maturation while using the Chambers protocol. The Anatomic protocol did not show the presence of non-neuronal cells as shown in Fig. 4B at DIV35.

**Fig. 5:**
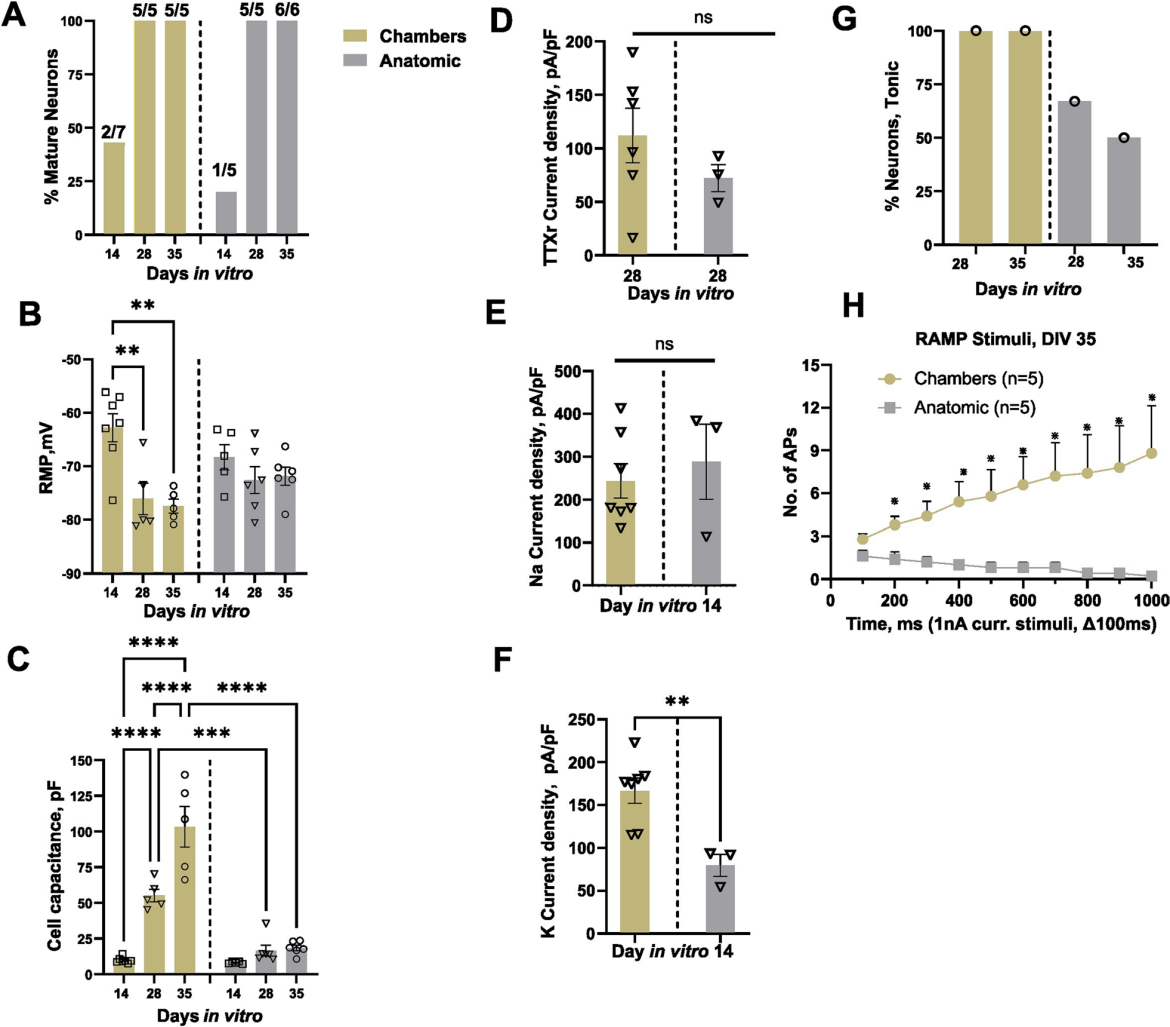
Comparison of electrophysiological characteristics of Anatomic and Chambers-derived neurons (Ctrl1). **A** Comparison of percentage of mature APs (First AP generated with a square pulse current injection) Percentage Mature neurons- DIV14: 43% (n = 2 out of 7), DIV28: 100% (n = 5 out of 5) and DIV35: 100% (n = 5 out of 5) for neurons generated with Chambers protocol. Number of cells having APs with overshoot above 0 mV out of total cells patched are denoted for each recording day above the bar. **B** RMP measured from both the protocols. One way Anova Bonferroni's multiple comparisons test. **C** Cell capacitance (pF) measured from both the protocols. One way Anova Bonferroni's multiple comparisons test. **D** TTXr currents recorded in the presence of 500 nM TTX. Mann–Whitney test. **E**, **F** Voltage clamp recordings of inward Na^+^ and K^+^ current density measured on DIV14 of maturation. Mann–Whitney test **G** Tonic firing neurons in response to ramp current stimuli. Chambers protocol- DIV28: n = 4 (4/4 Tonic, 1 cell no AP), DIV35: n = 5 (5/5 Tonic). **H** The average number of APs generated in response to ramp current stimuli. n = 5 for both protocols. Multiple t test. DIV-Days in vitro. Data are shown as mean ± SEM

The neurons generated by the Chambers protocol expressed peripherin and Tuj1 from DIV11 onwards (Fig. 4C). Current and voltage clamp recordings showed mature APs starting from DIV14. At DIV28, all recorded neurons showed APs with an overshoot above 0 mV, similar to neurons generated with the Anatomic protocol (Fig. 5A). The RMP was found to be more depolarized at DIV14 as compared to DIV28 and DIV35 for the Chambers protocol. We did not detect a significant difference over the same time span with the Anatomic protocol (Fig. 5B and Table 5). We observed an increase in cell size with maturation from both protocols and neurons with the Chambers protocol displaying significantly larger cell capacitance (Fig. 5C and Table 5). TTXr current density measured in the presence of TTX (500 nM) suggest the presence of Nav1.8 ion channels in these neurons on DIV28 with no significant difference in current density (pA/pF) compared to those neurons generated by the Anatomic protocol (Chambers DIV28: 72.2 ± 12.7, n = 3 vs Anatomic DIV28: 111.9 ± 25.4, n = 6; *p* = 0.38) (Fig. 5D). With physiological solutions, voltage clamp recordings revealed no significant difference in Na^+^ current density (Fig. 5E and Table 6), but we found significantly decreased K^+^ current density for neurons differentiated with the Anatomic protocol (Fig. 5F and Table 6). Tonic firing was predominant in the Chambers protocol (Fig. 5G).

**Table 5:** AP parameters—Chambers versus Anatomic

Parameters	Ctrl1 (Chambers)			Ctrl1 (Anatomic)		
	DIV14	DIV 28	DIV 35	DIV 14	DIV 28	DIV 35
RMP, mV	− 62.8 ± 2.6 (7)	− 76.1 ± 3.0^a^ (5)	− 77.4 ± 1.3^b^ (5)	− 68.3 ± 2.3 (5)	− 72.6 ± 2.5 (6)	− 71.9 ± 1.7 (6)
Cell capacitance, pF	9.8 ± 0.8^c^ (7)	55.2 ± 4.4 ^d^ (5)	103.3 ± 14.3^e^ (5)	8.0 ± 0.3 (5)	16.5 ± 3.8^f^ (6)	18.3 ± 1.9^g^ (6)

Data as Mean ± SEM

^a,^^b^*p* < 0.05 versus DIV 14 Ctrl1 (Chambers)

^c,^^d^*p* < 0.05 versus DIV 35 Ctrl1 (Chambers)

^e^*p* < 0.05 versus DIV 14 Ctrl1 (Chambers)

^f^*p* < 0.05 versus DIV 28 Ctrl1 (Chambers)

^g^*p* < 0.05 versus DIV 35 Ctrl1 Chambers)

The values in brackets indicate the sample number, DIV—Days in vitro

**Table 6:** Sodium and potassium currents Chambers versus Anatomic

Parameters	Ctrl1 (Chambers)	Ctrl1 (Anatomic)
	DIV14	DIV14
Na^+^ current density, pA/pF	243.7 ± 40.19 (7)	288 ± 87.5 (3)
K^+^ current density, pA/pF	166.8 ± 14.7* (7)	79.5 ± 12.8 (3)

Data as Mean ± SEM

*p < 0.05 versus DIV 14 Single cell Ctrl1 (anatomic)

The values in brackets indicate the sample number, DIV—Days in vitro

Ramp stimuli also resulted in increased neuronal firing with the Chambers protocol at DIV35 (Fig. 5H), possibly due to robust K^+^ channel expressions: we have observed that neurons on DIV14 from Chambers protocol show significantly higher K^+^ current density as compared to the Anatomic protocol. Both methods resulted in the differentiation and production of mature electrically-active sensory neurons from DIV28.

### Differentiation of iPSCs from pain patients

One of the significant advantages of iPSC-derived sensory neurons is to understand physiological changes that occur in the disease. We utilized the Anatomic protocol to differentiate iPSCs from two different pain syndromes: SFN and IEM.

### Electrophysiological phenotyping of SFN and IEM nociceptors displayed reduced AP rheobase and hyperexcitability

With an efficient generation of sensory neurons from Ctrl1 and Ctrl2 subjects using Anatomic protocol, we next examined the phenotype of iPSC-derived neurons from pain patients for disease modelling. We pooled the data from single cell and clump seeding for Ctrl1 iPSC-derived sensory neurons as we did not find any differences in their functional properties (Fig. 2). All the patch clamp data for disease modelling for Ctrl1, SFN, and IEM subjects were collected from DIV35-40. All three groups were differentiated once to generate sensory neurons.

Immunofluorescence staining confirmed the expression of peripherin and the pan-neuronal marker Tuj1 at DIV8, 14, and 28 for SFN-derived neurons and at DIV39 for IEM-derived neurons (Additional file 1: Fig. S6A-B). Voltage clamp recordings indicate the presence of TTXr currents in IEM and SFN patient-derived neurons (Additional file 1: Fig. S7).

**Fig. 6:**
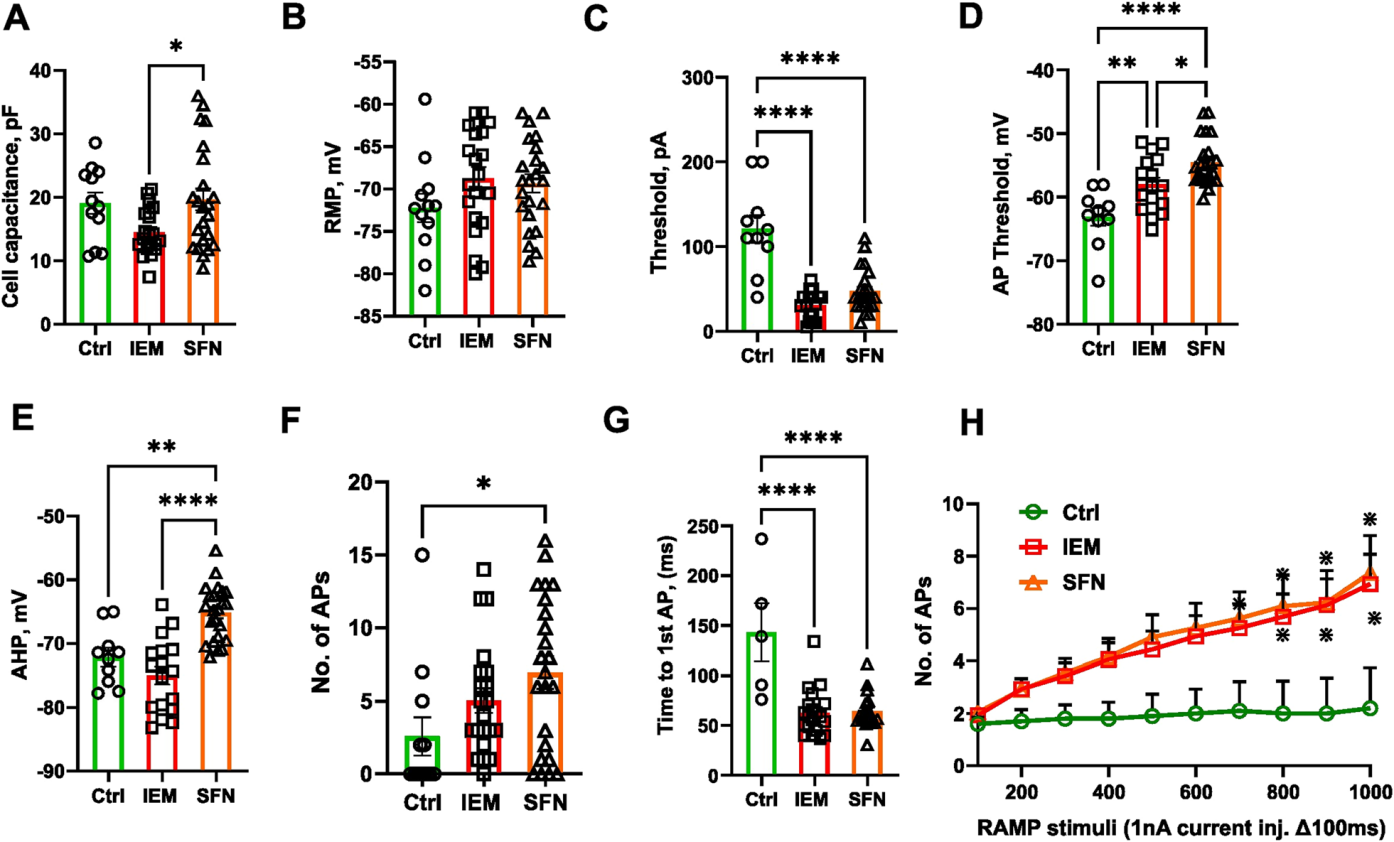
Disease modelling of IEM and SFN pain disorders using the Anatomic protocol. **A**, **B** Cell size and RMP did not show significant differences observed for both IEM and SFN groups as compared to control. **C** Rheobase indicate significant lesser current injection needed to generate an AP for both SFN and IEM group. **D** AP threshold shows significant shift to depolarized potentials for IEM and SFN-derived neurons as compared to control.** E** Significant shift of afterhyperpolarization (AHP) to more depolarized potentials for SFN patient as compared to control. **F** Ramp current stimuli 500 pA/500 ms resulted in a significant increase in no. of APs firing from SFN-derived neurons as compared to control group. **G** Time needed to generate 1st AP is significantly reduced for both IEM and SFN-derived neurons as compared to control. **H** In response to ramp current injections, there was a significant increase in number of APs fired from both patient-derived neurons (Ctrl n = 10, IEM n = 16, SFN n = 22). Two way ANOVA. **A**–**G** One way ANOVA with Tukey's multiple comparison test. Data are shown as mean ± SEM

To assess the neuronal excitability and AP parameters from iPSC-derived sensory neurons, we performed current-clamp recordings. Cell capacitance and RMP did not show significant difference in both patient-derived neurons as compared to control group (Fig. 6A, B and Table 7). We found that the patient sensory neurons require significantly less current injection to generate APs compared to the control group (Fig. 6C and Table 7). There was a significant difference between AP threshold of the patient-derived neurons as compared to the control group (Fig. 6D and Table 6). AHP was much more depolarized for the SFN patient-derived neurons compared to the control group (Fig. 6E and Table 7).

**Table 7:** AP characteristics of Ctrl1, IEM and SFN neurons

Parameters	Ctrl1	n	IEM	n	SFN	n
RMP, mV	− 72.2 ± 1.7	12	− 68.8 ± 1.3	21	− 69.3 ± 1.1	23
Cell capacitance, pF	19.1 ± 1.7	10	14.5 ± 0.8	17	19.6 ± 1.7	23
Threshold, pA	121 ± 16.3	10	31.1 ± 3.9*	17	47.4 ± 5.3*	23
AP threshold, mV	− 63.1 ± 1.4	10	− 58.0 ± 1.0*	17	− 54.5 ± 0.8*	23
AHP, mV	− 72.1 ± 1.4	10	− 75.0 ± 17.0	17	− 65.1 ± 0.9*	23
No. of APs (RAMP stimuli)	2.6 ± 1.3	12	5.0 ± 0.8	21	7.0 ± 1.1*	23
Time to 1st AP, ms	143.1 ± 29.0	5	63.0 ± 5.1*	20	64.5 ± 4.0*	19

Data as Mean ± SEM

*p < 0.05

We found a significant increase in the number of APs generated in response to ramp current stimuli (500 pA/500 ms) compared to control group (Fig. 6F and Table 7). Patient-derived neurons require significantly less time to generate the first AP in response to slow ramp depolarizations compared to control group indicating increased excitability for both IEM and SFN patient-derived neurons (Fig. 6G and Table 7). Diseased sensory neurons showed a significant increase in the number of APs fired, indicative of hyperexcitability in response to slower ramp depolarizations (Fig. 6H). We did not observe spontaneously firing neurons with SFN-derived neurons although our recording protocol limited the observation time to only 4 s. One of 16 IEM-derived neurons showed spontaneous firing. Altogether, electrophysiological phenotyping showed characteristics of elevated excitability in IEM and SFN-derived nociceptors.

### Nociceptor marker expression and functional characterization of anatomic protocol with RealDRG™

In the previous sections of this paper, we described and compared two differentiation protocols: Chambers and Anatomic using in house iPSC clones of healthy controls and patients. iPSC culture and differentiation is tedious and many research laboratories do not have the means to perform this complex research. An accessible alternative are pre-differentiated cells, which are commercially available. We used the Anatomic “RealDRG™” as they are very similar to those utilized in the previous sections since all the differentiation agents are identical. The only substantive difference is that the RealDRG™ cells are frozen in a pre-mature state and finally differentiated in the laboratory where the study takes place. Thus, we set out to perform a multi-center in-depth analysis of the RealDRG™ to assess their function and mRNA expression levels.

We assessed RealDRG™ for nociceptor marker expression using in situ hybridization and their functional profiling with membrane potential assays, automated patch clamp and multi-well microelectrode arrays.

### In situ hybridization of RealDRG™ neurons demonstrates correlation to human DRG gene expression

**Fig. 7:**
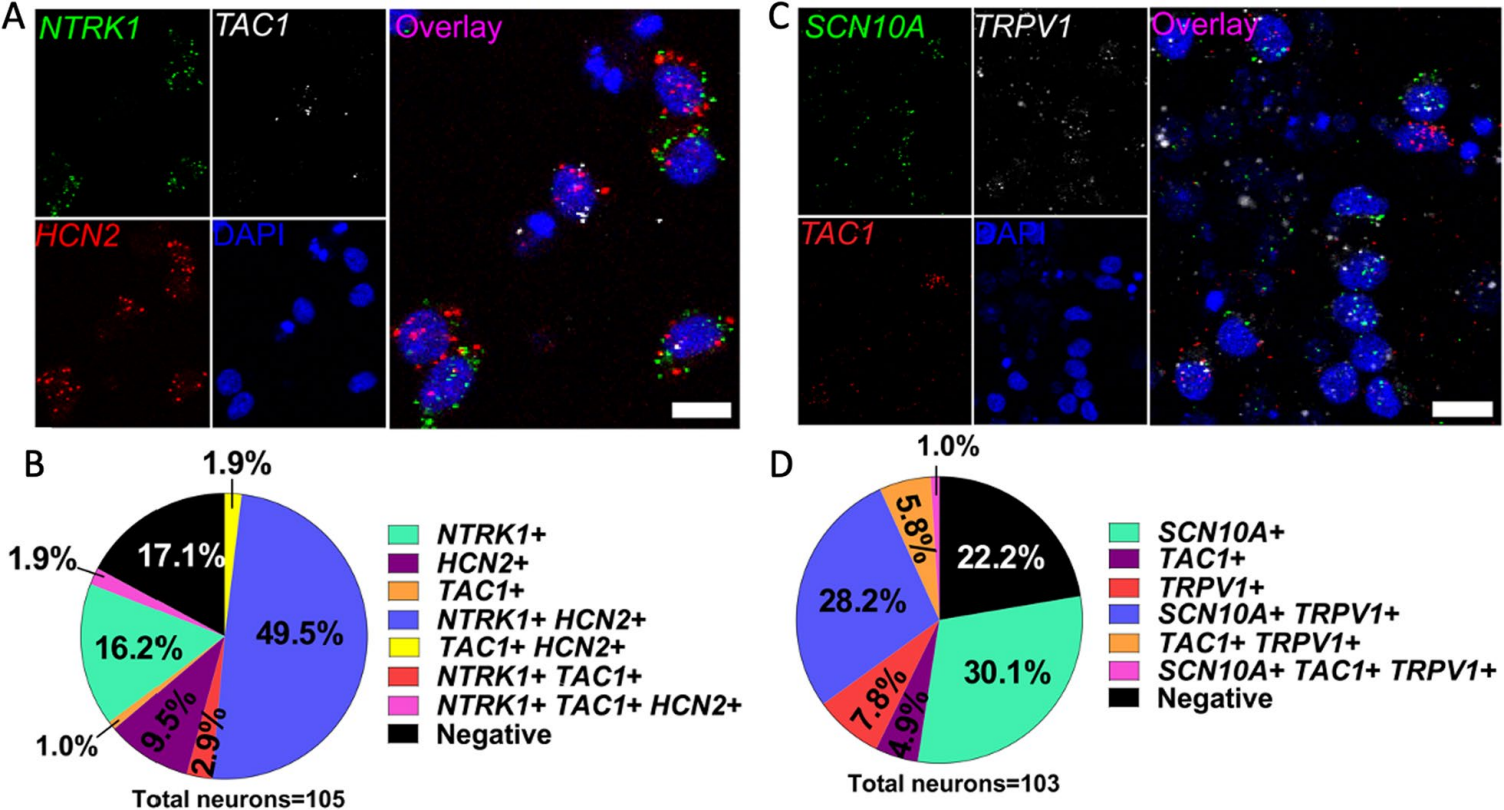
Gene expression in Anatomic RealDRG™ neurons. **A** Expression of mRNA for NTRK1 (TRKA, green), TAC1 (preprotachykinin-1; white) and HCN2 (red) seen in Anatomic protocol cells at DIV14 co-labeled with DNA marker DAPI (blue). **B** Pie chart for staining in **A** showing distributions of cells expressing indicated markers. **C** Expression of mRNA for SCN10A (Na_V_1.8, green), TRPV1 (white), TAC1 (red) seen in Anatomic protocol cells at DIV16 co-labeled with DNA marker DAPI (blue). **D** Pie chart for staining in **C** showing distributions of cells expressing indicated markers. Images are cropped from 40X images. Scale bar—10 µm

**Fig. 8:**
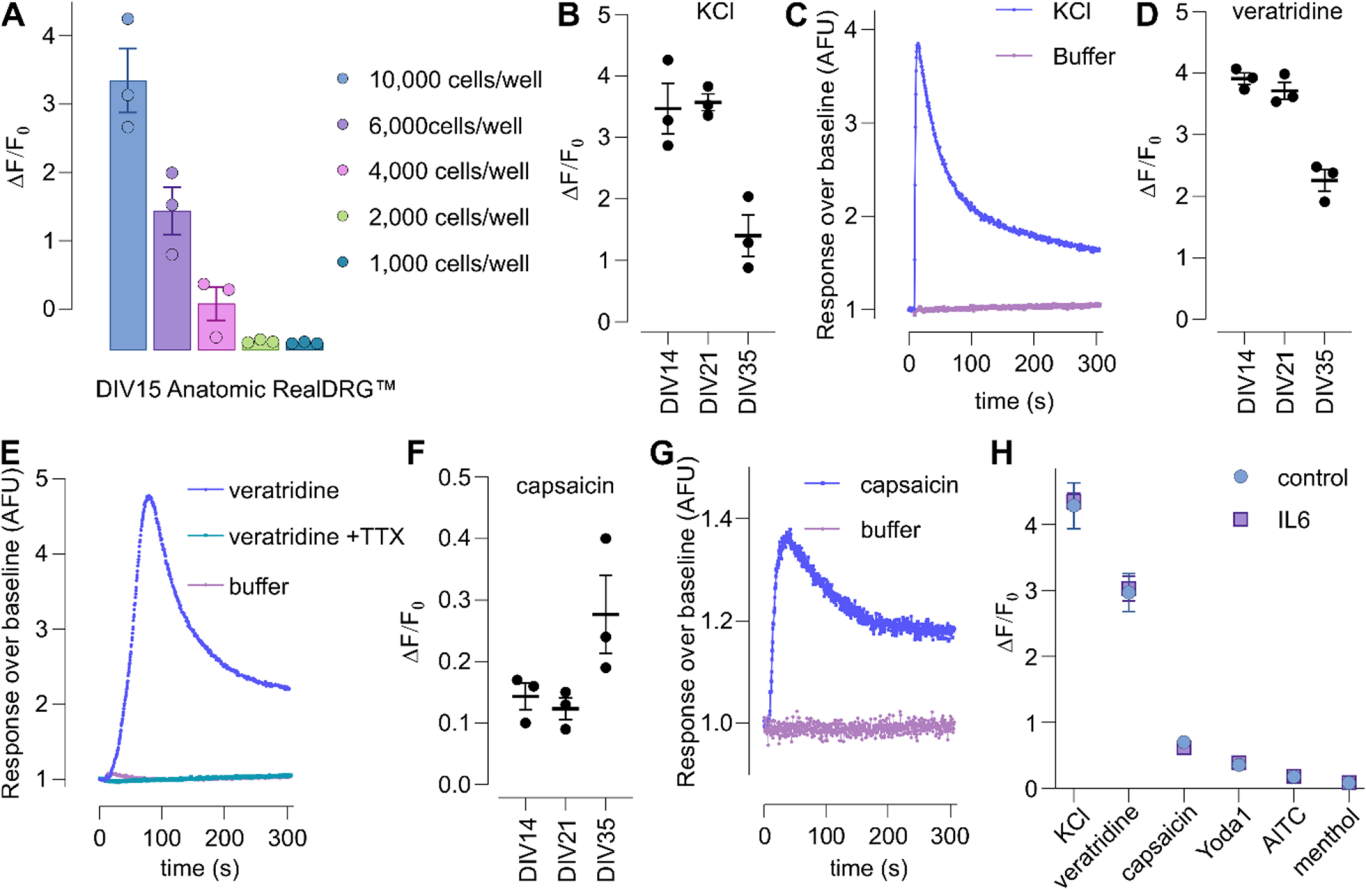
Anatomic RealDRG™ can be adapted for FLIPRPenta high-throughput Ca^2+^ assays. **A** Ca^2+^ responses of sensory neurons at DIV15 plated with varying cell densities (1000–10,000 cells/well) on 384-well plates. **B** KCl-induced Ca^2+^ responses at DIV15, DIV21 and DIV35 **C** sample trace from DIV21. **D** Veratridine-induced Ca^2+^ responses at DIV15, 21 and 35 **E** sample trace showing complete inhibition of responses by TTX (1 µM). **F** Capsaicin-induced Ca^2+^ responses emerge at DIV35, **G** capsaicin sample trace. **H** 24 h treatment with IL6/soluble IL6-R does not affect the response properties of sensory neurons at DIV35. Data are presented as mean ± S.D. from n > 3 replicates

To evaluate the expression of common human DRG nociceptor markers, multi-plex in situ hybridization was completed using the RNAscope protocol [32]. Two separate experiments were completed on RealDRG™ Anatomic Protocol neurons ~ 1 week after plating (DIV14 and 16). In the first experiment, mRNA for *NTRK1*, *TAC1*, and *HCN2* were probed at DIV14 (Fig. 7A, B). In the second experiment, mRNA for *SCN10A, TAC1*, and *TRPV1* were probed at DIV16 (Fig. 7C, D). All of these genes are highly expressed in human DRG neurons recovered from organ donors [38]. Over 80% of the RealDRG™ expressed NTRK1 and HCN2. Importantly, almost 60% of the Anatomic cells expressed SCN10A, a marker of nociceptors. Many of the RealDRG™ (42.8%) also expressed TRPV1 consistent with capsaicin responses seen in Fig. 8F, G and heat sensitivity seen in Fig. 10. TAC1, which is a pre-proprotein that codes for neurokinin A and substance P, was expressed in 7.7% and 11.7% of these cells on DIV14 and DIV16, respectively, demonstrating consistent expression between these two time points, but far lower expression than seen in human DRG neurons [39]. Sensory neurons generated with Chambers protocol has been reported to show expression of Substance P and CGRP indicating the presence of a subset of peptidergic nociceptors including upregulation of key nociceptor markers SCN9A, SCN10A, and SCN11A, the purinergic receptor P2RX3 and the vanilloid receptors TRPV1 and TRPM8 [6, 22, 24].

### FLIPR Ca^2+^ assays show that RealDRG™ respond to a variety of nociceptor stimuli in a 384-well format

To evaluate if RealDRG™ might also be amenable to high-throughput fluorescence assays, we first optimised seeding density in a 384-well format and assessed KCl depolarization-induced Ca^2+^ responses. As commonly observed for fluorescence assays, higher seeding densities resulted in enhanced signal-to-noise ratios, with 10,000 cells/well performing best (Fig. 8A). We next compared responses to the voltage activated Na^+^ channel activator veratridine (50 µM), the TRPV1 agonist capsaicin (1 µM) and KCl-induced depolarisation in iPSC-derived sensory neurons differentiated using the Anatomic protocol for DIV14, DIV21, and DIV35. Interestingly, KCl- and veratridine-induced responses were similar at DIV15 and DIV21 but decreased in magnitude at DIV35 (Fig. 8B–E), possibly due to the emergence of larger cell clusters following prolonged in vitro differentiation that may interfere with dye loading, while capsaicin-induced responses could only be observed at DIV35 but not at earlier timepoints (Fig. 8F, G). Veratridine-induced responses were inhibited completely by TTX (1 µM; Fig. 8E), consistent with the relatively larger TTX-s component observed in patch-clamp studies, as well as the resistance of the TTX-r Na_V_1.8 to modulation by veratridine [40].

We also compared Ca^2+^ responses in RealDRG™ neurons differentiated for DIV35 following treatment with IL-6 (100 ng/mL) and soluble IL-6 receptor (100 ng/mL) for 24 h (Fig. 8H), but observed no difference in response to either KCl (Control ΔF/F_0_ 4.28 ± 0.77, IL6 ΔF/F_0_ 4.35 ± 0.30; *p* > 0.05), veratridine (50 µM; Control ΔF/F_0_ 2.97 ± 0.57, IL6 ΔF/F_0_ 3.03 ± 0.49; *p* > 0.05), capsaicin (1 µM; Control ΔF/F_0_ 0.70 ± 0.09, IL6 ΔF/F_0_ 0.62 ± 0.12; *p* > 0.05), or the Piezo1 agonist Yoda1 (100 µM; Control ΔF/F_0_ 0.36 ± 0.08, IL6 ΔF/F_0_ 0.38 ± 0.14; *p* > 0.05). No response was observed to the TRPM8 activator menthol (1 mM; ΔF/F_0_ 0.08 ± 0.01) or the TRPA1 activator allyl isothiocyanate (AITC, 1 mM; ΔF/F_0_ 0.18 ± 0.07; Fig. 8H).

### RealDRG™ activity can be assessed by automated patch clamp recordings

**Fig. 9:**
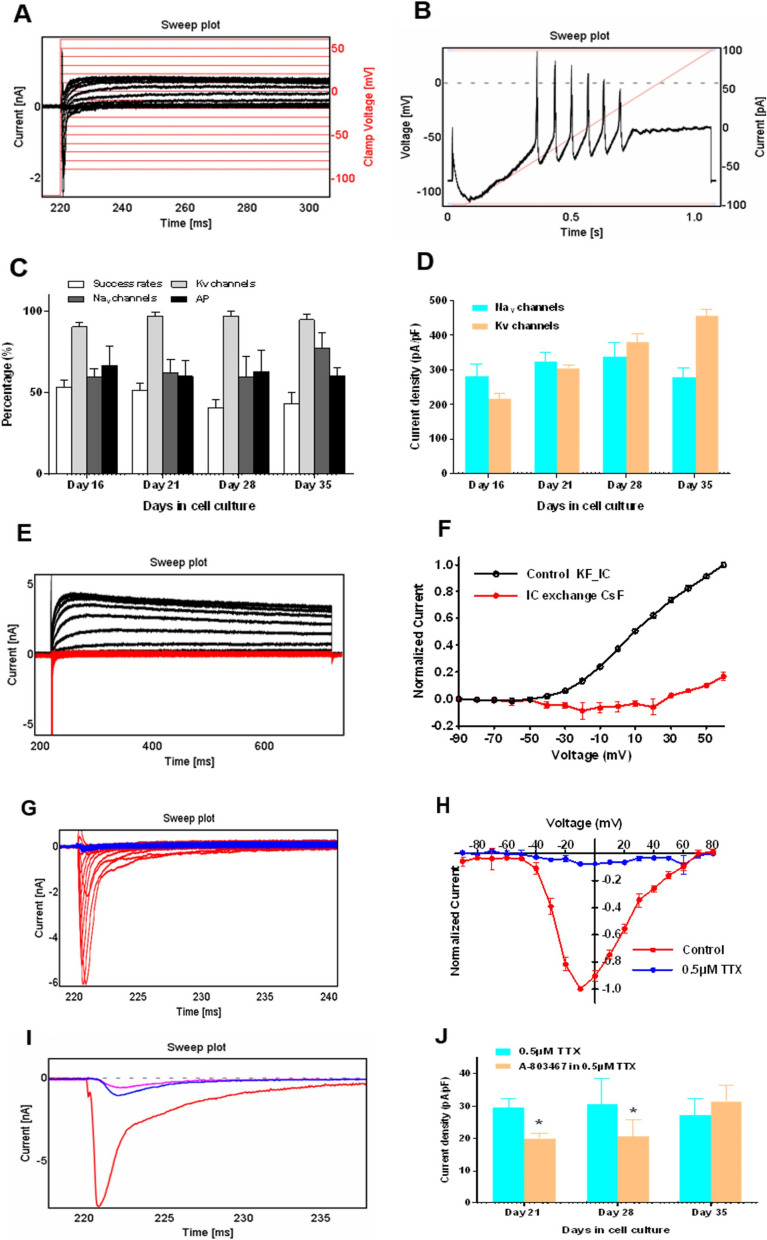
Voltage- and current-clamp recordings of Anatomic RealDRG™ on Qube384. **A** Representative current traces with holding voltage at − 90 mV. **B** Current-clamp recording: holding voltage at − 90 mV, ramp current clamp was elicited by injecting current from − 100 pA to 100 pA with 500 or 1000 ms duration. **C**, **D** Qube384 experiment success rates and current expression levels during culture period of 28 days. **C** Success rate indicates all the cells passed the criteria of R_mem_ > 200 MΩ, the rates were decreased with longer culture period, which are 54 ± 5% (n = 3), 52 ± 5% (n = 3), 41 ± 5% (n = 4), and 43 ± 7% (n = 4), respectively, for DIV16, 21, 28, and 35 days. For the cells passed membrane resistance and cell size filters, average expression levels of Kv_,_ Nav channels, and AP firings are 90–97%, 60–77%, and 60–67%, respectively. **D** Current densities were measured at depolarizations to − 10 mV, 20 ms for Nav currents_,_ and + 60 mV, 500 ms for Kv currents. **E** Current traces with internal solution of KF (black) and after exchanging to CsF based internal solution (red). Currents were elicited by 10 mV stepwise voltage increasing from − 90 to + 60 mV for 300 ms.** F** Current–voltage relationship curve were all normalized to the current amplitudes at + 60 mV before IC exchange. **G** Family of current traces in control and 0.5 µM TTX groups by using the same voltage protocol in Fig. 1A. **H** Current–voltage relationship curves, all the current amplitudes were normalized to the control currents at voltage of − 10 mV (Data showed DIV21 cells). **I** Current traces at voltage − 10 mV for control (red), 0.5 µM TTX (blue), and 10 µM A-803467 in 0.5 µM TTX (Magenta). **J** Current densities of 0.5 µM TTX and 10 µM A-803467 in 0.5 µM TTX for DIV21, 28, and 35

RealDRG™ were characterized for functional expression of Na^+^ and K^+^ ion channels using APC. Representative voltage clamp and current clamp recordings are shown in Fig. 9A, B. We analysed the Qube384 experiment success rates and current expression levels of iPSC-derived sensory neurons during the maturation period. Success rate was defined as percentage of the cells passing the criteria of R_mem_ > 200 MΩ. We found that the success rates for the experimental runs were decreased with longer culture period, 54 ± 5% (n_Qchips_ = 3), 52 ± 5% (n_Qchips_ = 3), 41 ± 5% (n_Qchips_ = 4), and 43 ± 7% (n_Qchips_ = 4), respectively, for DIV16, DIV21, DIV28, and DIV35, respectively (Fig. 9C).

For the cells passing the membrane resistance and cell size filter criteria, average expression levels of voltage gated K^+^ and Na^+^ channels, and AP firings were 90–97%, 60–77%, and 60–67%, respectively (Fig. 9C). We examined the Na^+^ and K^+^ current densities measured at depolarization potential to –10 mV, applied for 20 ms_,_ and to + 60 mV applied for 500 ms. No significant difference was observed for Na^+^ current density (pA/pF) 282 ± 36 (n = 42), 323 ± 30 (n = 103), 339 ± 40 (n = 46), and 279 ± 26 (n = 125) for DIV16, 21, 28, and 35, respectively (Fig. 9D). However, K^+^ current densities increased with the increasing days of culture (pA/pF), 216 ± 16 (n = 55), 305 ± 10 (n = 138), 378 ± 27 (n = 68), and 457 ± 17 (n = 163) at DIV16, 21, 28 and 35, respectively (Fig. 9D). We measured current–voltage relationship curves for K^+^ and Na^+^ currents. Current traces with internal solution of KF (black) and after exchanging to CsF based internal solution (red) are shown in Fig. 9E. Current–voltage relationship curve were all normalized to the current amplitudes at + 60 mV before internal solution exchange (Fig. 9F). Representative current traces in control and after application of 0.5 µM TTX are shown in Fig. 9G. Current–voltage relationship for control and after TTX application are shown in Fig. 9H at DIV21 of maturation.

We further evaluated the effect of selective Nav1.8 blocker A-803467 at DIV21, 28 and 35 in the presence of 0.5 µM TTX application. Representative current traces evoked by a voltage of -10 mV are shown for control, 0.5 µM TTX, and 10 µM A-803467 (Fig. 9I). Current densities of 0.5 µM TTX and 10 µM A-803467 in 0.5 µM TTX for DIV21, 28 and 35 are shown in Fig. 9J. We found a significant reduction in current densities (pA/pF) at DIV21 and 28 after application of 10 µM A-803467 + 0.5 µM TTX, (30 ± 3 vs 20 ± 2 (n = 36) and 31 ± 8 vs 21 ± 5 (n = 12), respectively, for DIV21 and 28). However, at DIV35, A-803467 failed to show an effect (27 ± 5 vs 32 ± 5 pA/pF (n = 18) Fig. 9J.

### Multi-well microelectrode arrays demonstrate the spontaneous activity and temperature sensitivity of RealDRG™

**Fig. 10:**
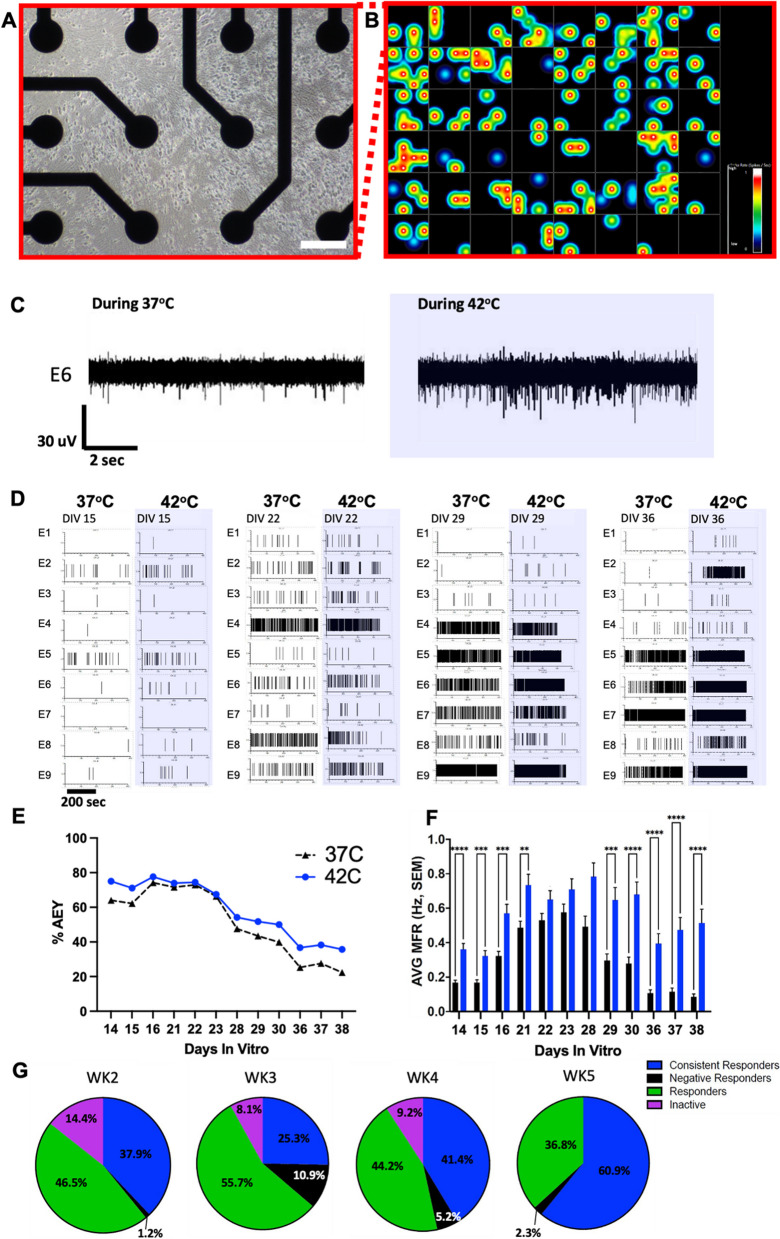
MEA recordings of Anatomic RealDRG™ sensory neurons over the maturation period. **A** Cells were plated over the electrode array region within a typical well of the Axion 48 well cytoview MEA plate shown here at DIV16 (16 electrodes/well). Scale bar—100 µm. **B** Data visualization from RealDRGs cultured on MEAs. Heat plot across the 48 well MEA indicating spike activity. Each colored circle is an electrode with detected APs. **C** Representative traces showing a single active electrode before and during a 42 °C temperature ramp at DIV36. **D** Representative raster plots tracking the per-electrode responsiveness to 42 °C heat ramp from a single timepoint each during week 2, 3, 4, and 5. **E** The percent active electrode yield (AEY) from 384 electrodes in a 48-well plate for three recording sessions each during week 2, 3, 4, and 5 after plating of cells. Blue trace describes the AEY during 42 °C trials, the 37 °C electrode data (prior to heating) is shown in black. **F** The average Mean Firing Rate (MFR, Hz, mean ± SEM) for 384 electrodes in a 48-well plate for three recording sessions each during week 2, 3, 4, and 5 after plating of cells. Blue bars describe the MFR during 42 °C trials at each timepoint, the 37 °C trials MFR is shown in black. Asterisks denote significance with Sidak’s multiple comparisons (**p < 0.01, ***p < 0.001, ****p < 0.0001). **G** Pie charts describing the evolution of responsiveness to 42 °C from the 174 active electrodes identified at week 5 from their response in the previous weeks. The four types of responses during week 5 were: consistent (positive response from 3 of 3 42 °C trials/wk), responders (1 ≤ positive trials < 3 trials/wk), negative (spontaneously active with negative responses to 42 °C), and inactive electrodes (< 1 spk/min)

Anatomic protocol RealDRG™ were grown on Axion 48-well (16 electrodes/well) MEAs (Fig. 10A, B). We documented the mean firing rate (MFR) per electrode during each data acquisition session for three days each week for four weeks total starting on DIV14. The active electrode yield (AEY) was calculated based on electrodes whose MFR was 1 spike/min or higher per session. RealDRG™ cells respond robustly to 37-to-42 °C temperature changes during all recording sessions (DIV36) (Fig. 10C) consistent with functional TRPV1 receptors which respond to this temperature range. Within a single well (16 total microelectrodes), we observed a variety of firing patterns that change across the 4 weeks in vitro (Fig. 10D). AEY ranged from 77.6 to 22.4% with a mean ± SEM of 55.2 ± 3.7% (n = 384 microelectrodes) with distinct patterns (Fig. 10E). AEY tended to decrease over time but was stable during the three recording sessions each week. We found that the average MFR from all microelectrodes ranged from 0.08 to 0.78 Hz with a mean ± SEM of 0.43 ± 0.04 Hz with the highest and most stable patterns of activity seen during the 4th and 5th week recording sessions (Fig. 10F). Looking at the impact of the 42 °C temperature ramp on modulating the MFR at each DIV, there was a significant main effect of temperature (2-WAY ANOVA, *p* < 0.0001; main effect of temperature x DIV *p* < 0.0001) with Sidak’s multiple comparisons tests showing significant differences between baseline and heated at most of the time points (Fig. 10F). Additionally, there was a significant effect of DIV time (2-WAY ANOVA, main effect of DIV *p* < 0.0001). A closer look suggested changes in baseline MFR (“pre-heat”) both between weeks and during each week’s three consecutive recordings. The difference within weeks was most apparent for weeks 2–4 but stabilized within the week 5 recording sessions. When classifying the cells by week, we found some notable patterns (Fig. 10G). First, most cells responded to the temperature ramp early after plating (week 2). Furthermore, the proportion of cells that responded to the temperature ramp was fairly consistent across weeks and appeared to increase by week 5. For example, during week 4 the combination of “consistent responders” and “responders” (see Methods) reached 85.6%. By week 5, this sum of “responders” and “consistent responders” significantly increased to 97.7% (*p* < 0.0001, t-test).

Altogether these results indicate that RealDRG™ sensory neurons can be used for ion channel/receptor characterization and drug screening purposes using high throughput methods.

## Discussion

In this study, we compared the utility of two differentiation methods of human iPSCs into peripheral neurons for disease modelling. Present differentiation methods of iPSCs into sensory neurons show differences in the efficacy and clone-to-clone variations raising reproducibility concerns. Here we compared two methods to derive sensory neurons from iPSCs. The Anatomic method involved an accelerated primal ectodermal lineage within 24 h of differentiation [26], resulting in mature functional neurons by DIV28 without the use of mitomycin C to suppress non-neuronal cell growth, and the second one, the Chambers method [6], used small molecule differentiation protocol for generation of sensory neurons within a comparable time frame resulting in a more heterogenous cell population. We further characterized the function and expression of key nociceptor markers and hence their utility for screening novel compounds using automated high throughput platforms.

Conventional differentiation protocols require a long maturation period of 8–10 weeks to yield mature sensory neurons for disease modelling [22, 41, 42]. While the Anatomic protocol produced a purer cell culture, the duration required for maturation was similar to that needed for the Chambers protocol. We performed a detailed functional characterization of two control and two cell lines from pain patients with the Anatomic protocol and compared them with the Chambers protocol for one of the control cell lines. We found that both Anatomic and Chambers protocols resulted in neurons with mature AP characteristics from DIV28 of maturation and expression of TTXr currents from DIV14 onwards indicating presence of nociceptive-like neurons (Fig. 2A, D). Pharmacological evaluation indicated the presence of TTXr currents by a selective Nav1.8 blocker (Fig. 2E). We also found that K^+^ current density was significantly lower in Anatomic protocol on DIV14 as compared to Chambers protocol, which may have contributed to the higher tonic firing observed with Chambers protocol (Fig. 5H). We also observed increased K^+^ current density with maturation using automated patch clamp recordings (Fig. 9D). We found that both protocols generate mature sensory neurons with peripheral neuronal identity at about the identical time point. We find a striking difference in the expression of voltage activated K^+^ channel function indicating a higher tonic firing observed with neurons from the Chambers protocol. Future work will assess the subtypes of sensory neurons produced by both differentiation protocols.

We investigated multiple iPSC cell lines derived from different reprogramming methods, genetics, age, and somatic cell source. We found that with the Anatomic protocol, all iPSC lines resulted into the generation of dense immature neuronal network with fewer non-neuronal cells within DIV7 compared to 10–14 days with the conventional Chambers differentiation protocol. The Anatomic protocol capitalizes on inhibiting selected signaling pathways enabling the immediate exit from pluripotency and converts the stem cells within 24 h into precursor cells that can be directed to generate neural populations [26] based on the production of an OCT4-negative/PAX6-negative population after 24 h of incubation with small molecule inhibitors as compared to day 5 with the chambers protocol. This is followed by the generation of neural crest cells and immature neurons by day 4 and day 7 of differentiation, respectively (Fig. 1B, C). CD271, also known as LNGFR (low-affinity nerve growth factor receptor), NGFR (nerve growth factor receptor), or p75 NTR (neurotrophin receptor), belongs to the tumor necrosis factor receptor superfamily and is important for development, survival, and differentiation of neural cells. Umehara et al. [10] showed a robust expression of p75 and human natural killer 1 (HNK1) double positive cells indicating a high efficiency of neural crest induction for generation of peripheral sensory neurons.

We implemented MACS sorting for p75 marker at DIV10 of Chambers differentiation to obtain a more pure neuronal culture. However, one of the control clones still showed non-neuronal cell growth in the culture after MACS sorting (Fig. 4A). Morphologically, the Anatomic protocol does not yield a substantial amount of detectable non-neuronal cells with four different cell lines tested for generation of sensory neurons (Fig. 1B, C, 3A, B). The Anatomic protocol was suitable for differentiating Ctrl2, the “resistant clone”.

We established the generation of functional sensory neurons with Anatomic protocol having mature AP properties from DIV28 of maturation. With an efficient generation of sensory neurons from the clones Ctrl1 and Ctrl2 (resistant clone) using Anatomic protocol, we next examined the phenotype of iPSC-derived sensory neurons from pain patients for disease modelling. We investigated sensory neurons derived from two patients suffering from IEM and SFN disorders. Cellular excitability of SFN patient-derived nociceptors displayed a significantly increased excitability: they showed more spikes and more active electrodes when recorded with MEAs [25]. We found a significant reduction in the current threshold for the patient-derived neurons compared to control group (Ctrl:121 ± 16.3 pA, IEM: 31.1 ± 3.9, SFN: 47.4 ± 5.3 *p* < 0.05) (Fig. 6C). In addition, we found a similar hyperexcitability phenomenon to evoked stimuli with manual patch clamp recordings in response to ramp stimuli (Fig. 6H). IEM neurons derived by the Anatomic protocol also displayed higher excitability to electrical stimuli as reported with the Chambers protocol (Fig. 6H). The AP threshold remained unaltered in SFN-derived nociceptors with Chambers protocol [25]. It has been reported that L554P/Nav1.8 mutation causing SFN disorder results to significant reduction in current threshold after expression of L554P mutation in DRG neurons and no change in RMP was observed [43]. Similarly, the pathogenic variant described in SCN10A (c.5116A > G) identified in a pain patient revealed that mutant channels decrease current threshold and increase the firing frequency of evoked APs when transfected into small rodent DRG neurons [44]. We found similar results for SFN-derived neurons using Anatomic protocol.

C-fibers of patients suffering from neuropathic pain are spontaneously active [45]. SFN patient-derived neurons did not display spontaneous firing as reported earlier. Namer et al. [25] detected a larger number of spontaneously active SFN sensory neurons (19.4%) compared to control (1.8%). We did not observe spontaneous firing of neurons differentiated by the Anatomic protocol. This might be due to a longer RMP recording (60 s was used immediately after obtaining whole cell configuration) as compared to our recordings with 4 s at RMP. We observed only one cell with spontaneous firing from IEM patient-derived neurons which was 6.25% of the total cells patched. Anatomic protocol may require longer maturation period and additional growth factors to express robust ion channels to mimic spontaneous firing neurons observed for both the patient-derived neurons.

Recently Recently Saito-Diaz et al. [46] reported the generation of three sensory neuronal subtypes-nociceptors, mechanoreceptors, and proprioceptors by day 20 of maturation at a ratio of approximately 2:1:1, correlating with the relative sensory neuronal distribution in the human DRG in vivo [47]. It is possible that subtype specific neurons generated with Anatomic protocol might not represent the molecular diversity of DRG sensory neurons found in vivo although many of our results suggest that some important nociceptor subtypes are represented. Further molecular characterization of iPSC nociceptors with single cell sequencing should be considered for identification of specific neuronal subtypes and comparison to existing human nociceptor datasets [39, 48]. Altogether, electrophysiological phenotyping shows characteristics of higher frequency firing in IEM and SFN nociceptors for both differentiation protocols.

Here, RealDRG™ were further characterized for sensory neuron phenotype using RNAscope, FLIPR, Automated patch clamp and Multielectrode arrays. In situ hybridization of RealDRG™ demonstrated both evidence of heterogeneity in the Anatomic cells and similarity between Anatomic protocol cells and human primary nociceptors. Heterogeneity of nociceptive markers in Anatomic cells indicates that the cells may be useful as a model to explore human DRG physiology as a whole. We find evidence for good, although not perfect, expression patterns in the Anatomic protocol RealDRG™ cells compared to human DRGs (data available on request). The majority of neurons express SCN10A and many of those also express TRPV1 (~ 50%), similar to human neurons, although with a smaller population that is TRPV1-positive (human DRG ~ 75% TRPV1) [38]. Only 15–20% of neurons generated with Chambers protocol were responsive to capsaicin [22]. Also consistent with human DRG neurons [38], most RealDRGs express NTRK1, which is a major difference that distinguishes human nociceptors from mouse nociceptors where only half of the cells express NTRK1 [39]. HCN2 expression was similar to what is observed in the native human DRG [49]. While these preliminary findings require further investigation to characterize these populations more thoroughly, the existing data suggest that the neurons derived from the Anatomic protocol can be binned into actions of particular subsets of neurons that may be informative in making decisions about human nociceptors. It has been also reported that sensory neurons generated with Chambers protocol demonstrates expression of key nociceptor sensory markers P2RX3, TRPM8, TRPV1, SCN9A and SCN10A [6, 22, 24].

A major advantage of human iPSC-derived sensory neurons is the ability to generate, at least in principle, unlimited numbers of cells, which opens the possibility of adapting these cellular model systems to high-throughput drug discovery efforts. In FLIPR^Penta^ assays adapted to 384-well format, we observed particularly robust depolarisation-induced responses that are likely mediated by voltage-gated Ca^2+^ channels. In contrast, responses to capsaicin were relatively small and would likely require further optimisation of assay conditions. Given the tendency of cells differentiated for more than 4–5 weeks to form cell clusters, this could include bulk differentiation and subsequent dissociation/replating for high-throughput assays, similar to our approach for automated patch-clamp electrophysiology. While we did not observe functional responses to menthol or AITC (Fig. 8H), suggesting that Anatomic iPSC-derived sensory neurons represent a subset of the functionally diverse types of DRG neurons found in primary culture, our transcriptomic analysis suggests that these cells can be adapted for functional assays assessing responses of a range of therapeutically relevant targets. In addition to FLIPR assay for high throughput recordings, we have shown functional recordings of Na^+^ and K^+^ channels in RealDRG™ with automated patch clamp recordings (Fig. 9). We observed success rates of > 40% over the maturation period starting DIV16, 21, 28, and 35 days (Fig. 9C). The success rate decreased with increasing maturation indicating dense neuronal network formation of iPSC-derived neurons making it difficult to obtain single cells after dissociation. We also observed TTXr currents in these neurons at DIV21, 28 and 35 of maturation (Fig. 9J). Finally, we utilized MEAs to evaluate firing of Anatomic protocol RealDRG™ across time and responses to heat ramp stimuli. MEAs in plate form provide a valuable read-out (i.e., firing) for drug screening efforts. We find that RealDRG™ are spontaneously active with good stability during a single week and variability in active yield between weeks. For drug screening this would indicate that testing could occur within a week in a single plate but that week-to-week comparisons might be more difficult to interpret. The RealDRG™ respond to temperature ramps, again indicating the functional presence of heat activated channels such as TRPV1. From a drug screening perspective, such phenotypic responses of RealDRG™ would be useful for distinguishing between drugs that inhibit all sensory neurons versus only those cells with heat sensitivity.

Taken together all three automated/semi-automated platforms provide a valuable approach in characterizing various ion channels from iPSC-derived sensory neurons to facilitate drug discovery and development of novel targets.

## Conclusions

In summary, we demonstrate that both Anatomic and Chambers protocols may have the potential to be utilized for disease modelling and pharmacological investigations. The findings described here represent a step forward toward development of a iPSC nociceptor-based approach to personalized medicine for painful neuropathies.

### Supplementary Information

**Additional file 1**. Supplementary information.

## Data Availability

The authors confirm that the data supporting the findings of this study are available within the article and its supplementary material.

## Abbreviations

iPSCs: Induced pluripotent stem cells
NCCs: Neural crest cells
TTXr: Tetrodotoxin resistant
TTXs: Tetrodotoxin sensitive
RMP: Resting membrane potential
AP: Action potential
PNS: Peripheral nervous system
DRG: Dorsal root ganglion
IEM: Inherited erythromelalgia
SFN: Small fiber neuropathy
DIV: Days in vitro
PBMCs: Peripheral blood mononuclear cells
MACS: Magnetic activated cell sorting
AHP: Afterhyperpolarization
ECS: Extracellular solution
APC: Automated patch clamp
Rm: Membrane resistance
SEM: Standard error of mean
MEA: Multi-well microelectrode arrays
MFR: Mean firing rate
ESCs: Embryonic stem cells
AEY: Active electrode yield
NTR: Neurotrophin receptor
FLIPR: Fluorescent Imaging Plate Reader
HCN2: Hyperpolarization-activated cyclic nucleotide-gated ion channel 2
LNGFR: Low-affinity nerve growth factor receptor
NGFR: Nerve growth factor receptor
HNK1: Human natural killer 1
