# Supplementary material for: How to differentiate induced pluripotent stem cells into sensory neurons for disease modelling: a functional assessment
Source: Stem Cell Res Ther. 2024 Apr 5;15:99. doi: 10.1186/s13287-024-03696-2 (PMC10998320; doi:10.1186/s13287-024-03696-2)
Supplement: Supplementary file 1 — Additional file 1. Supplementary information. [file 13287_2024_3696_MOESM1_ESM.docx]

**Supplementary Figures:**

**How to differentiate induced pluripotent stem cells into sensory neurons for disease modelling: a functional assessment**

Anil Kumar Kalia^1,2^, Corinna Rösseler^1^, Rafael Granja-Vazquez^3,4^, Ayesha Ahmad^5^, Joseph J. Pancrazio^4^, Anika Neureiter^1^, Mei Zhang^6^, Daniel Sauter^6^, Irina Vetter^7,8^, Asa Andersson^7^, Gregory Dussor^3,5^, Theodore J. Price^3,5^, Benedict J. Kolber^3,5^, Vincent Truong^9^, Patrick Walsh^9^, Angelika Lampert^1,2,10*^

Affiliations

^1^Institute of Neurophysiology, Uniklinik RWTH Aachen University, Pauwelsstr. 30, 52074 Aachen, Germany

^2^Research Training Group 2416 MultiSenses-MultiScales, RWTH Aachen University, Aachen, Germany

^3^Center for Advanced Pain Studies, University of Texas at Dallas, Richardson, TX 75080, USA

^4^Department of Bioengineering, University of Texas at Dallas, Richardson, TX 75080, USA

^5^Department of Neuroscience, University of Texas at Dallas, Richardson, TX 75080, USA

^6^Sophion Bioscience Inc., Bedford, MA 01730, USA

^7^Institute for Molecular Bioscience, The University of Queensland, St Lucia, QLD 4072, Australia

^8^School of Pharmacy, The University of Queensland, Woolloongabba, QLD 4102, Australia

^9^Anatomic Incorporated, 2112 Broadway Street NE #135, Minneapolis, MN 55413

^10^Scientific Center for Neuropathic Pain Aachen - SCN^Aachen^, Uniklinik RWTH Aachen University, 52074 Aachen, Germany

*Corresponding author: alampert@ukaachen.de

**Fig.S1** Immunofluorescence staining of iPSC-derived sensory neurons from Ctrl2 with Chambers protocol.

1.
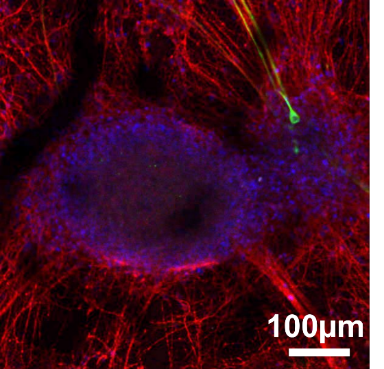

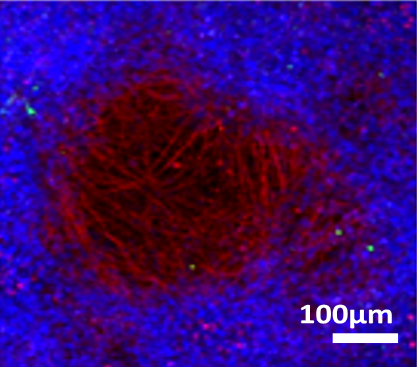
 B.

Neurons only show neuronal staining for Tuj1 marker protein and fail to express peripherin. A. DIV30 and B. DIV60. Scale Bar 100µm, DIV-Days *in vitro*. Peripherin- green, Tuj1-red and DAPI-blue fluorescence.

**Fig.S2** Sanger sequencing confirms presence of heterozygous CAG to GAG conversion (c.2623) of Nav1.7 in IEM patient cells resulting in glutamine to glutamic acid substitution (p.Q875E/Nav1.7).


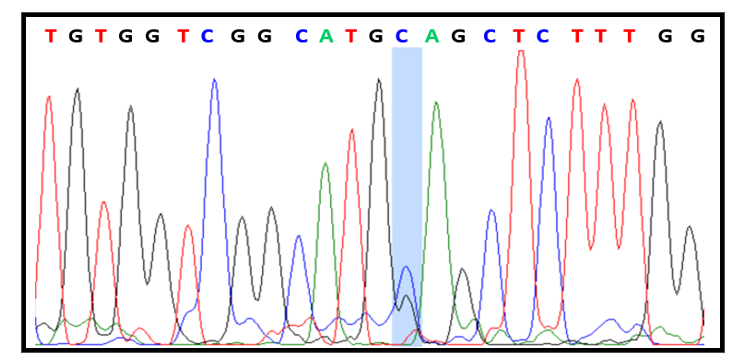


**Fig.S3** Phase contrast images of iPSCs from Ctrl1, Ctrl2, IEM and SFN subjects display typical pluripotent-like cell morphology. Scale bar 1000µm.


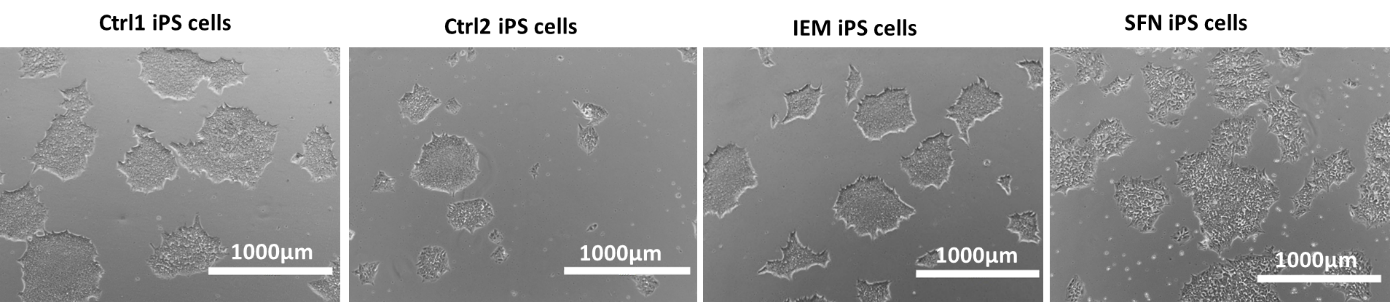


**Fig.S4** Phase contrast images display single cell seeding and clump seeding of iPSCs (Ctrl2) on DIV0 of differentiation and generation of immature neurons by DIV7. Scale bar 1000µm and 200µm.


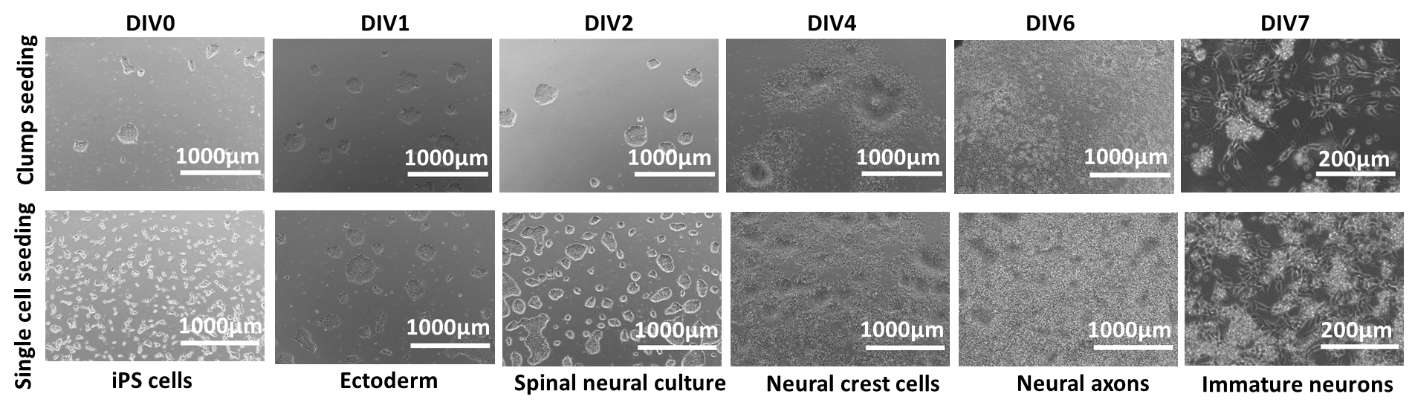


**Fig.S5**. Voltage clamp recordings: TTXs and TTXr components of sodium channels (DIV31, clump seeding method). i. Voltage pulse protocol ii. Representative voltage clamp recordings with total sodium currents, TTXs and TTXr components. TTXr currents were further blocked by 1µM A-887826.


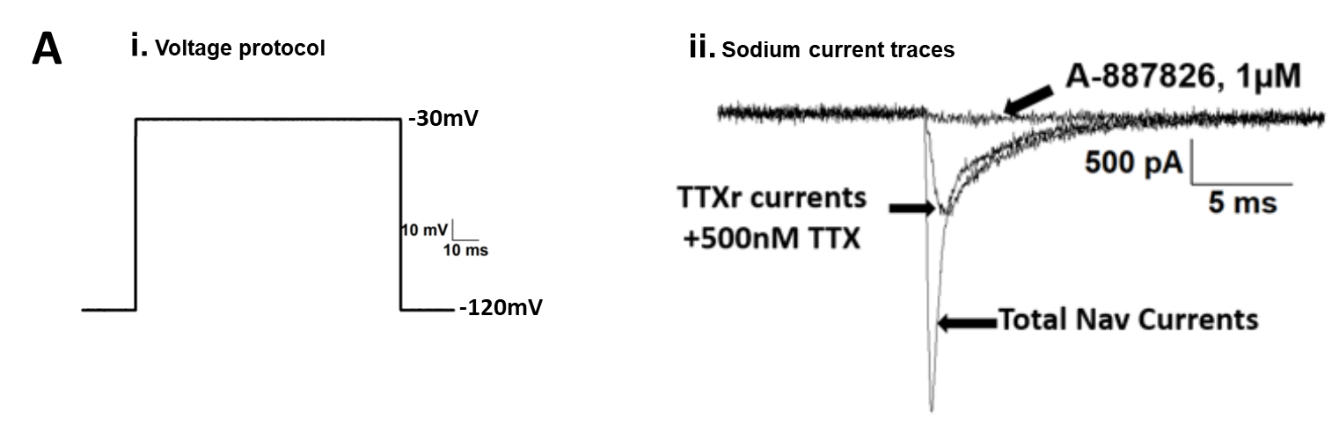


**Fig.S6** Immunofluorescence staining of iPSC-derived neurons from IEM and SFN patients using the Anatomic protocol.

**A.** IEM patient-derived neurons confirm peripheral neuronal identity at DIV39. **B.** Peripheral neuronal identity conformed with Peripherin and Tuj1 staining at DIV8,14 and 28 for SFN- derived neurons. Scale Bar DIV8 and 14 - 100µm, DIV-Days *in vitro*. Peripherin-green, Tuj1-red and DAPI- blue fluorescence. Scale Bar indicated for each picture.

**
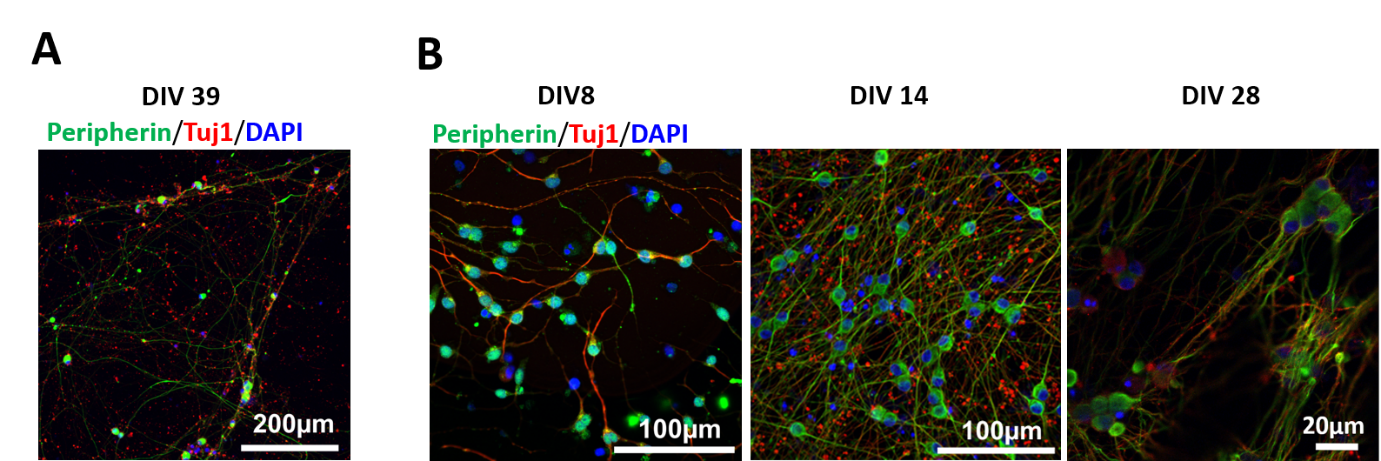
**

**Fig.S7** Electrophysiological characterization of iPSC-derived neurons from IEM and SFN patients. TTXr current density measured on DIV14, 21, 35 for SFN and DIV35 for IEM-derived neurons. One way ANOVA with Tukey's multiple comparison test. Data are shown as mean ± SEM.
